# In-feed bambermycin medication induces anti-inflammatory effects and prevents parietal cell loss without influencing *Helicobacter suis* colonization in the stomach of mice

**DOI:** 10.1186/s13567-018-0530-1

**Published:** 2018-04-10

**Authors:** Chloë De Witte, Bernard Taminiau, Bram Flahou, Veerle Hautekiet, Georges Daube, Richard Ducatelle, Freddy Haesebrouck

**Affiliations:** 1 Department of Pathology, Bacteriology and Avian Diseases, Faculty of Veterinary Medicine, Ghent University, Merelbeke, Belgium; 2 Department of Food Sciences, FARAH, Université de Liège, Avenue de Cureghem 10, 4000 Liège, Belgium; 3 Huvepharma, Uitbreidingstraat 80, 2600 Antwerp, Belgium

## Abstract

The minimum inhibitory concentration of bambermycin on three porcine *Helicobacter suis* strains was shown to be 8 μg/mL. The effect of in-feed medication with this antibiotic on the course of a gastric infection with one of these strains, the host response and the gastric microbiota was determined in mice, as all of these parameters may be involved in gastric pathology. In *H. suis* infected mice which were not treated with bambermycin, an increased number of infiltrating B-cells, T-cells and macrophages in combination with a Th2 response was demonstrated, as well as a decreased parietal cell mass. Compared to this non-treated, infected group, in *H. suis* infected mice medicated with bambermycin, gastric *H. suis* colonization was not altered, but a decreased number of infiltrating T-cells, B-cells and macrophages as well as downregulated expressions of IL-1β, IL-8M, IL-10 and IFN-γ were demonstrated and the parietal cell mass was not affected. In bambermycin treated mice that were not infected with *H. suis*, the number of infiltrating T-cells and expression of IL-1β were lower than in non-infected mice that did not receive bambermycin. Gastric microbiota analysis indicated that the relative abundance of bacteria that might exert unfavorable effects on the host was decreased during bambermycin supplementation. In conclusion, bambermycin did not affect *H. suis* colonization, but decreased gastric inflammation and inhibited the effects of a *H. suis* infection on parietal cell loss. Not only direct interaction of *H. suis* with parietal cells, but also inflammation may play a role in death of these gastric acid producing cells.

**Electronic supplementary material:**

The online version of this article (10.1186/s13567-018-0530-1) contains supplementary material, which is available to authorized users.

## Introduction

*Helicobacter suis* is a Gram-negative, spiral-shaped bacterium found in the stomach of up to 90% of pigs at slaughter age, causing gastritis and a decreased daily weight gain [1, 2]. In addition, infection with this bacterium has been associated with ulceration of the non-glandular part of the porcine stomach, most likely through induction of gastric acid secretion alterations and by influencing the gastric microbiota [3]. Apart from pigs, *H. suis* is the most prevalent gastric non-*H. pylori Helicobacter* (NHPH) species in humans. *H. suis* infection in humans has been associated with gastritis, peptic ulcers and mucosa-associated lymphoid tissue (MALT) lymphoma [4]. Direct or indirect contact with *H. suis* infected pigs or consumption of contaminated water or raw or undercooked pork may be a source of human infection [5]. Since rodent models are easily colonized with *H. suis*, the role of wild mice as vectors might also be considered.

In general, *Helicobacter* sp. infected human patients are treated with a combination of a proton pump inhibitor and two or three antibiotics selected from clarithromycin, amoxicillin, metronidazole, tetracycline and/or levofloxacin [6]. A similar therapeutic protocol is not indicated in *H. suis* infected pigs since this is expensive, labor intensive and antibiotic use may favor spread of antimicrobial resistance in pathogens as well as in bacteria belonging to the microbiota [7]. In addition, no vaccine formulation is available which completely protects pigs against *H. suis* infection [8], making development of an alternative therapy desirable [4]. Recently, an inhibitory action of bambermycin, a glycolipid antibiotic, has been described towards *H. pylori* strains [9]. As *H. pylori* is closely related to *H. suis*, it can be hypothesized that bambermycin might inhibit *H. suis* as well.

Bambermycin (synonyms: flavophospholipol, moenomycin, flavomycin) disrupts bacterial cell wall synthesis [10] and is mainly active against Gram-positive bacteria and to some extent against Gram-negative bacteria such as *Pasteurella* sp., *Brucella* sp. and *H. pylori* [10]. Due to its high molecular weight, bambermycin is not absorbed in the gastro-intestinal tract after oral administration. Until 2006, it was used as a growth promoter in animal feed in the European Union [10]. The growth promoting effects of bambermycin supplementation have been linked with a better equilibrium of the gastro-intestinal microbiota which might be due to a reduced colonization of pathogens, such as *Salmonella enterica*, *Clostridium perfringens* and *Fusobacterium* spp. [10–13], while bacteria considered to have beneficial effects, such as *Lactobacillus* spp., are not affected. The effect on *S. enterica* and *C. perfringens* in the intestinal tract, however, contrasts with the relative insensitivity of these species to bambermycin in vitro [10]. Although bambermycin was frequently used as a feed additive for over 50 years, acquired resistance, transfer of resistance or cross-resistance with other antimicrobials has not yet been reported [10]. It has been described that bambermycin selectively inhibits the growth of *Escherichia coli* and *S. enterica* harboring resistance plasmids and decreases the conjugation transfer frequency of resistance plasmids in *E. coli*, *S. enterica* and *Staphylococcus aureus* [14–16].

The main objective of this study was to determine the effect of bambermycin supplementation on the course of a *H. suis* infection, the host response and the gastric microbiota, as all these parameters may be involved in gastric pathology. BALB/c mice were used as an experimental model, as in previous studies we showed that these rodents can easily be colonized with *H. suis,* resulting in gastritis, epithelial cell hyperproliferation and necrosis of parietal cells [17], which are also representing the main characteristics of a human and porcine infection with this agent.

A second objective of our study was to determine if in-feed medication with bambermycin might be useful to control *H. suis* infections in pigs.

## Materials and methods

### Minimum inhibitory concentration (MIC) of bambermycin on *H. suis* strains

#### *H. suis* strains

*Helicobacter suis* strains HS1, HS8 and P13/26 were isolated from the gastric mucosa of pigs from different herds according to the method described by Baele et al. [18]. The strains were shown to be genetically different by multilocus sequence typing [19].

All strains were cultured under biphasic and microaerobic conditions at 37 °C. The biphasic medium consisted of Brucella agar (BD, Franklin Lakes, New Jersey, USA) supplemented with 20% fetal calf serum (GE Healthcare Life Sciences, Logan, USA), 5 mg amphotericin B/l (Sigma-Aldrich, Saint Louis, Missouri, USA), Campylobacter selective supplement (Oxoid) and Vitox supplement (Oxoid). The pH of the agar was adjusted to 5 by adding HCl to a final concentration of approximately 0.05%. Finally, Brucella broth (BD, pH 5) was added on top. Isolates were passaged twice to ensure reliable growth. After incubation, the bacteria were harvested and the concentration was determined using an improved Neubauer counting chamber (Sigma-Aldrich).

#### Determination of MIC-values of bambermycin

The MIC of bambermycin for *H. suis* strains HS1, HS8 and P13/26 was determined according to the method described by Vermoote et al. [20]. In brief, a combined Brucella agar and broth dilution method in 24-well plates was used. Twofold dilutions of bambermycin (flavomycin^®^, Huvepharma, Antwerp, Belgium) were added to the agar and broth, with final concentrations ranging from 0.03 to 128 μg/mL. In total, 5 × 10^7^ bacteria/mL were added to the broth and incubated during 48 h under microaerobic conditions. After incubation, *H. suis* was quantified using a quantitative real-time PCR (qPCR) where the *ureA* gene was amplified. The MIC was determined as the lowest concentration of bambermycin for which ∆C_t_ was at least 1 C_t_ higher than ∆cC_t_ (∆C_t_ = C_t_ after incubation − C_t_ before incubation of the bambermycin exposed strains; ∆cC_t_ = C_t_ after incubation − C_t_ before incubation of the controls; C_t_ = threshold cycle value) [20]. This is the lowest concentration of bambermycin with at least 50% less bacterial growth compared to controls without bambermycin.

*Staphylococcus aureus* ATCC 29213 was included as a reference strain. For this strain, 4 different MIC assays were performed: the broth microdilution procedure according to the CLSI standards, the method described by Butaye et al. [21, 22], the *H. suis* susceptibility assay conditions at pH 5 and the same conditions but at pH7.

### Effect of bambermycin supplemented diet on a *H. suis* infection in mice

#### Ethic statement

The in vivo experimental protocol was approved by the Ethical Committee of the Faculty of Veterinary Medicine, Ghent University, Belgium (EC 2015/131; November 17^th^, 2015).

#### Animals and experimental design

Forty-eight specific-pathogen-free (SPF) female, 5-week-old BALB/cOlaHsd mice were purchased from Harlan NL (Horst, the Netherlands). All animals tested negative for presence of *Helicobacter* spp. by the use of a *Helicobacter* genus specific PCR. The animals were randomly divided into 6 groups, each consisting of 8 mice. The groups were assigned as followed: non-*H. suis* infected mice fed a control diet without bambermycin (*H. suis*-negative control group; group 1), non-*H. suis* infected mice fed a 32 ppm bambermycin supplemented diet (group 2), non-*H. suis* infected mice fed a 64 ppm bambermycin supplemented diet (group 3), *H. suis* infected mice fed a control diet without bambermycin (*H. suis*-positive control group, group 4), *H. suis* infected mice fed a 32 ppm bambermycin supplemented diet (group 5) and *H. suis* infected mice fed a 64 ppm bambermycin supplemented diet (group 6). For each group, the mice were housed on autoclaved soft wood shavings in 2 separate filter-top cages to minimize cage-effect (i.e. four mice per cage). Drinking water was provided ad libitum. The animals were monitored several times a day during the whole experiment. Enrichment was provided in the form of paper tissues, mouse houses and other homemade available enrichment products. All animals were exposed to a 12:12 light:dark cycle in the same stable under controlled environmental conditions.

One week after arrival, groups 4–6 were inoculated twice with *H. suis* strain HS1 with a 48 h interval. Under brief isoflurane anesthesia and using a ball-tipped gavage needle, 350 μL Brucella broth (pH 5) containing 7 × 10^7^ bacteria of HS1 was administered intragastrically. Groups 1–3 received an equal volume of Brucella broth (pH 5). The mice were held in an upright position until they regained consciousness, to minimize the risk of reflux. Starting from 1 week after inoculation, the mice were fed ad libitum a control diet (groups 1 and 4), a diet supplemented with 32 ppm bambermycin (groups 2 and 5) or a diet supplemented with 64 ppm bambermycin (groups 3 and 6). The bambermycin supplemented diets were identical to the control diet, with the exception of being supplemented with 32 or 64 ppm bambermycin and with a variable amount of corn starch, used to correct minor differences in the energy content (Research Diets Inc., New Brunswick, USA). Finally, 9 weeks after the second inoculation, the mice were euthanized by cervical dislocation under deep isoflurane anesthesia. The stomachs were removed and opened along the major curvature. Four longitudinal strips were taken from the forestomach to the duodenum. One strip was fixed in 10% phosphate-buffered formalin and used for histopathology and immunohistochemistry. The other strips were used for DNA- and RNA-extraction.

#### Histopathology and immunohistochemistry

The formalin fixed longitudinal strip from the stomach was embedded in paraffin, sectioned at 5 μm, rehydrated and deparaffinized.

For each stomach, one of the sections was stained with haematoxylin and eosin, dehydrated and finally mounted with a coverslip for light microscopic evaluation. The severity of gastritis was scored according to the updated Sydney system with some modifications [1, 23]. Both diffuse infiltration with inflammatory cells and the presence of lymphoid aggregates and lymphoid follicles in the mucosa and submucosa were taken into consideration. The diffuse infiltration of mononuclear and polymorphonuclear cells was scored as follows: score 0 for absence of infiltration, score 1 for mild infiltration, score 2 for moderate infiltration and score 3 for marked infiltration. In addition, the formation of lymphoid follicles was scored as follows: score 0 for absence of lymphoid aggregates, score 1 for presence of a small number of lymphoid aggregates (*n* < 5), score 2 for a large number of lymphoid aggregates (*n* > 5) and/or the presence of 1 organized lymphoid follicle and score 3 for the presence of at least 2 organized lymphoid follicles. Based on the scoring of the diffuse infiltration with inflammatory cells and the presence of lymphoid aggregates and lymphoid follicles, an overall gastritis score was obtained. Therefore, the average score was calculated for each group. When an overall score of 0 < *n* ≤ 1; 1 < *n* ≤ 2 or 2 < *n* ≤ 3 was obtained, the gastritis was considered as mild, moderate and severe, respectively.

The other sections were used to determine *H. suis* colonization density, to study the presence of lymphoepithelial lesions, to analyze the number of infiltrating T-cells, B-cells and macrophages and, finally, to determine the number of parietal cells, necrotic cells and replicating cells. After rehydration and deparaffinization, heat-induced antigen retrieval was performed in citrate buffer (pH 6) using a microwave oven. Slides were incubated with 3% H_2_O_2_ in methanol (5 min) to block endogenous peroxidase activity and 30% goat serum (30 min) to block non-specific reactions. Negative controls to confirm the specificity of the secondary antibodies were obtained by incubating the sections without the primary antibodies.

*Helicobacter suis* colonization was visualized using a polyclonal genus-specific rabbit anti-*H. pylori* antibody (1/320; DakoCytomation, Glostrup, Denmark). For detection of infiltrating T- and B-cells, a polyclonal rabbit anti-CD3 antibody (1/100; DakoCytomation, Glostrup, Denmark) and a polyclonal rabbit anti-CD20 antibody (1/25; Thermo Scientific, Fremont, USA) were used, respectively. Incubation with primary antibodies directed against *Helicobacter*, CD3 and CD20 was followed by incubation with a biotinylated goat anti-rabbit IgG antibody (1/500; DakoCytomation). After rinsing, the sections were incubated with a streptavidin–biotin-HRP complex (Agilent Technologies, Santa Clara, California, USA) and the color was developed with diaminobenzidine tetrahydrochloride (DAB) and H_2_O_2_.

To determine the infiltrating macrophages, a primary antibody directed against the F4/80 surface marker (1/50; Santa Cruz Biotechnology, Santa Cruz, USA) was used. Detection was done using a rat ABC staining system (Santa Cruz Biotechnology). Apoptotic cells were identified by immunohistochemical staining using a rabbit polyclonal antibody directed against active caspase-3 and an anti-rabbit HRP-AEC cell and tissue staining kit (R&D Systems, Minneapolis, USA). Replicating cells were identified using a mouse monoclonal anti-Ki67 antibody (1/25; Novocastra Laboratories Ltd, Newcastle upon Tyne, UK) and a biotinylated goat anti-mouse IgG antibody (1/200; DakoCytomation). Subsequent visualization was done as described for CD3 and CD20 staining. Parietal cells were identified by immunohistochemical staining for the hydrogen potassium ATPase using a mouse monoclonal antibody (1/200; Abcam Ltd, Cambridge, UK) and a biotinylated goat anti-mouse IgG antibody (1/200; DakoCytomation). Subsequent visualization was done as described for CD3 and CD20 staining. A monoclonal mouse anti-cytokeratin antibody (1/50; DakoCytomation) was used to highlight lymphoepithelial lesions.

*Helicobacter suis* colonization density was scored according to the updated Sydney system [23]. T-cells, B-cells, macrophages, apoptotic cells, replicating cells and parietal cells were counted in five randomly chosen high power fields (magnification: 400×). The average number of positive cells per high power field was then calculated for each mouse.

#### Quantification of *H. suis* by real time PCR

DNA was extracted from the second gastric tissue strip using the Isolate II Genomic DNA Kit^®^ (Bioline, Taunton, USA), according to the instructions of the manufacturer. The presence of *H. suis* DNA was determined using a species-specific, quantitative real time (RT)-PCR based on the *ureA* gene [24]. The copy number of the obtained amplicons was calculated and converted to the number of *H. suis* bacteria per mg gastric tissue, by including Tenfold dilutions of an external standard consisting of a 1236 bp segment of the *ureAB* gene cluster from *H. suis* strain HS5, as described previously [25].

#### Expression of markers for inflammation and gastric acid secretion

RNA was extracted from the third gastric tissue strip using the RNeasy Mini Kit^®^ (Qiagen, Hilden, Germany), according to the manufacturer’s instructions. The obtained RNA concentrations were measured using a NanoDrop^®^ spectrophotometer (Isogen Life Science, Utrecht, The Netherlands), after which the concentration of each sample was adjusted to 1 μg/μL, followed by cDNA synthesis using the iScript™ cDNA Synthesis Kit (Bio-Rad, California, USA). Expression analysis was then performed for genes encoding host factors involved in gastric acid secretion (H+/K+ATPase, Sonic Hedgehog, KCNQ1, gastrin, the cholinergic muscarinic M3 receptor, somatostatin, the histamine H2 receptor and the gastrin CCK-B receptor) and in inflammation (IL-4, IL-6, IL-8, IL-10, IL-17, Il-1β, IFN-γ and TNFα). The housekeeping genes *PPIa*, *H2afz* and *HPRT* were shown to have a stable mRNA expression and therefore included as reference genes [26]. All primer sequences are shown in Additional file 1. The mRNA expression levels of the reference and target genes were quantified using a RT-PCR, as described earlier [26]. No-template-control reaction mixtures were included and all samples were run in duplicate. The C_t_-values were first normalized to the geometric mean of the C_t_-values of the reference genes. Fold changes were calculated using ΔΔC_T_ method with mean of C_t_-values from the control groups (groups 1 and 4). Finally, for each target gene, the results were expressed as fold changes of the mRNA expression of groups 2–4 and 5–6 relative to mRNA expression levels of the *H. suis*-negative control group and the *H. suis*-positive control group, respectively.

#### Gastric microbiota composition

DNA was extracted from the fourth gastric tissue strip using the DNeasy Blood & Tissue Kit^®^ (Qiagen) according to the instructions of the manufacturer. 16S rRNA amplicon pyrosequencing was performed using the Roche GS-Junior Genome Sequencer as described previously by Rodriguez et al. [27]. The obtained 16S rRNA sequence reads were processed using MOTHUR (software package v1.35), Pyronoise algorithm and UCHIME algorithm for alignment and clustering, denoising and chimera detection, respectively [28, 29]. The obtained read sets were compared to a reference dataset of aligned sequences of the corresponding region derived from the SILVA database (v1.15) of full-length rRNA sequences implemented in MOTHUR [30]. The final reads were clustered into operational taxonomic units (OTUs) using the nearest neighbor algorithm using MOTHUR with a 0.03 distance unit cutoff. A taxonomic identity was attributed to each OTU by comparison with the SILVA database using a 80% homogeneity cutoff. When a taxonomic identification lower than 80% was obtained, the taxonomic level was labelled with the first defined level from higher level followed by the label “unclassified”. Finally, the unique sequences for each OTU were compared with the SILVA data set using the BLASTN algorithm. For each OTU, a consensus taxonomic identification was given when less than 1% of mismatch with the aligned sequence was obtained. In the final metadata table, the following labelling was used: the population is identical to a taxonomically defined species and is labelled “genus_species”; the population is identical to a reference sequence belonging to a still undefined species and is labelled “genus_NCBI accession number”; the sequence is not identical to any known sequence and is labelled with the corresponding OTU number.

In order to determine the effect of *H. suis* and/or bambermycin supplementation on the gastric microbiota composition, different groups were compared with each other:

(i) *Helicobacter suis*-negative mice without bambermycin supplementation (i.e. group 1) (*n* = 5) vs *H. suis*-negative mice with bambermycin supplementation (i.e. groups 2–3) (*n* = 7), *H. suis*-positive mice without bambermycin supplementation (i.e. group 4) (*n* = 4) and *H. suis*-positive mice with bambermycin supplementation (*n* = 9) (i.e. groups 5–6).
(ii) *Helicobacter suis*-negative mice (i.e. groups 1–3) vs *H. suis*-positive mice (i.e. groups 4–6).
(iii) Mice without bambermycin supplementation (i.e. groups 1 and 4) vs mice with bambermycin supplementation (i.e. groups 2–3 and 5–6).

Subsampled datasets were obtained and evaluated in MOTHUR to estimate the richness, microbial diversity and population evenness by using the Chao1 estimator, Simpson’s reciprocal index and Simpson’s evenness index, respectively [31, 32]. Population structure and community membership were assessed with MOTHUR using distance matrix based on Bray–Curtis dissimilarity index. Differences in functional profiles of gastric bacterial communities were analyzed by mapping taxa into several phenotypes (i.e. metabolism, Gram staining, sporulation,…) using METAGENassist^®^ [33]. Only the phenotypes detected in more than 50% of the samples were included for further analysis.

#### Statistical analysis

Statistical analysis was performed using SPSS statistics 24^®^ (IBM, New York, USA). Differences in histopathology and fold changes of the markers for gastric acid secretion and inflammation were investigated using the non-parametric Kruskal–Wallis test with Bonferroni correction for multiple comparisons. Correlations between histopathology, fold changes and the number of *H. suis* bacteria were examined using the Pearson correlation coefficient. Differences were considered statistically significant at a corrected *P* value of less than 0.05.

Statistical differences in microbial diversity, richness and population evenness between the groups were investigated using non-parametric Kruskal–Wallis tests with Tukey post hoc tests using PRISM 7 (Graphpad Software). Using MOTHUR, community composition differences were investigated using Analysis of molecular variance (AMOVA) and homogeneity of molecular variance (HOMOVA). In order to highlight statistical differences in relative bacterial abundance between the groups, non-parametric Kruskal–Wallis tests with Tukey post hoc tests and Benjamini–Hochberg false discovery rate were performed using the STAMP software. Differences were considered statistically significant at a corrected *P* value of less than 0.05.

## Results

### MIC of bambermycin on *H. suis* strains

For all investigated *H. suis* strains, the MIC of bambermycin was 8 μg/mL. For the reference strain *S. aureus* ATCC 29213 the MIC-value was 0.12 μg/mL when tested according to the method described by Butaye et al. [21, 22] and 0.5 μg/mL when tested according to the CLSI standards, while MIC-values of 1 and 2 μg/mL were obtained using the *H. suis* susceptibility assay conditions at pH 7 and 5, respectively.

### Effect of bambermycin supplementation on *H. suis* colonization in mice

Non-*H. suis* infected groups 1, 2 and 3 tested negative for presence of *H. suis* as determined by qPCR and immunohistochemical staining, while all *H. suis* infected groups 4, 5 and 6 tested positive for *H. suis*. In both bambermycin-supplemented diet groups, the number of colonizing *H. suis* bacteria per mg gastric tissue as well as the colonization density was not altered compared to the *H. suis*-positive control group without bambermycin supplementation (Additional file 2). In all *H. suis* infected groups, *H. suis* bacteria were often found in the lumen of the gastric glands and in close proximity of parietal cells (Additional file 3).

### Effect of bambermycin supplementation on *H. suis*-infection associated pathologies in mice

#### Inflammation

**Figure 1:**
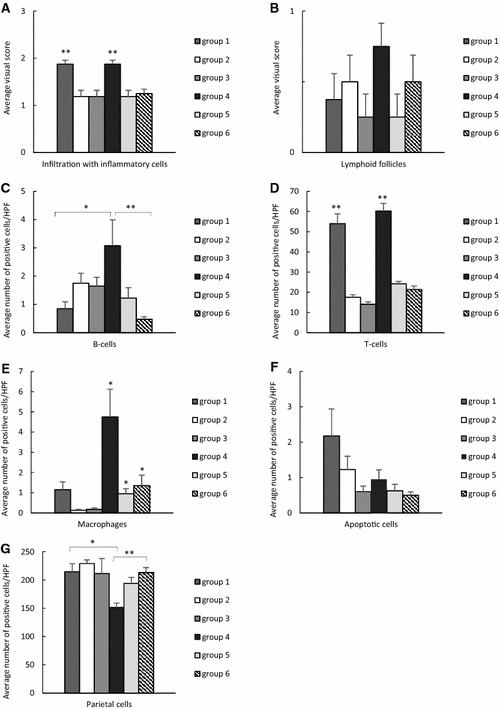
**Scores for immune cells, apoptotic cells and parietal cells in bambermycin supplemented and non-supplemented groups.** Scores for infiltration with inflammatory cells (**A**) and lymphoid follicle formation (**B**); number of infiltrating B-cells (**C**), T-cells (**D**), macrophages (**E**); number of apoptotic cells (**F**) and parietal cells (**G**) in bambermycin supplemented and non-supplemented groups. **A**, **B** Data are shown as the average scores for infiltration with inflammatory cells/lymphoid follicle formation, with standard deviation, of group 1–6. **C**–**G** Data are shown as the average number of positive cells per high power field (HPF), with standard deviation, belonging to a defined cell population, including B-cells, T-cells, macrophages, necrotic cells and parietal cells, of group 1–6. Statistical differences were calculated using the non-parametric Kruskal–Wallis H test SPSS statistics 24^®^. *Significant differences between the groups (*P* < 0.05), **significant differences between the groups (*P* < 0.005). Group 1 = *H. suis*-negative control group without bambermycin supplementation; group 2 = 32 ppm bambermycin supplemented, non-*H. suis* infected group; group 3 = 64 ppm bambermycin supplemented, non-*H. suis* infected group; group 4 = *H. suis*-positive control group without bambermycin supplementation; group 5 = 32 ppm bambermycin supplemented, *H. suis* infected group; group 6 = 64 ppm bambermycin supplemented, *H. suis* infected group.

An overview of the defined immune cell populations of each group is shown in Figure 1.

**Figure 2:**
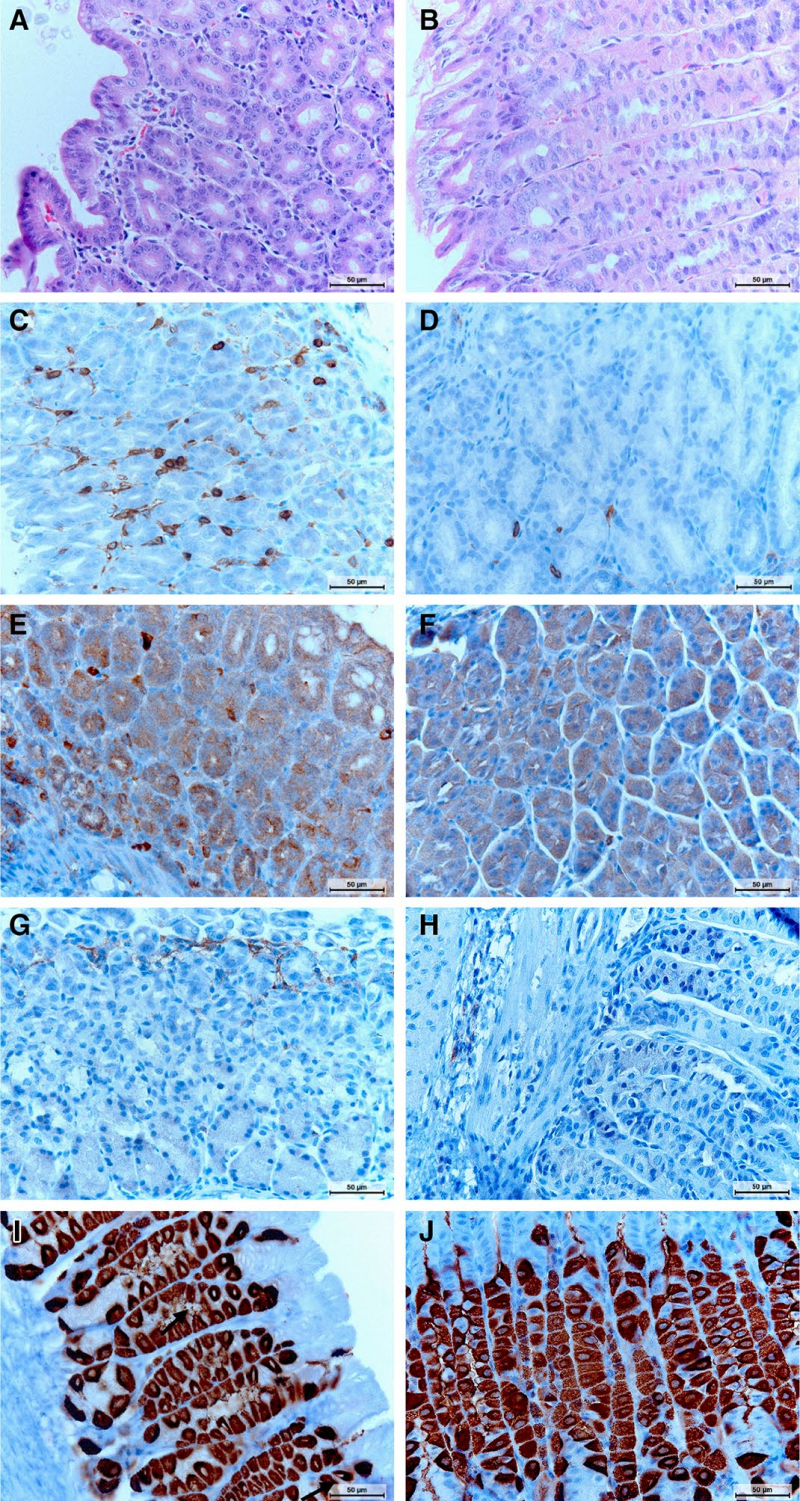
**Microscopic visualization of immune cells, apoptotic cells, proliferative cells and parietal cells in the mouse stomach. A**, **B** HE-staining showing infiltration with mononuclear and polymorphonuclear cells in *H. suis* infected mice without (**A**) and with bambermycin (**B**) supplementation. The infiltration is less severe during bambermycin supplementation. Original magnification: ×400. **C**, **D** CD3-staining showing infiltration with T-cells (brown) in *H. suis* infected mice without (**C**) and with bambermycin (**D**) supplementation. The infiltration is less severe during bambermycin supplementation. Original magnification: ×400. **E**, **F** CD20-staining showing infiltration with B-cells (dark brown) in mice infected with (**E**) and without *H. suis* (**F**). The infiltration is more severe during *H. suis* infection. Original magnification: ×400. **G**, **H** F4/80 staining showing infiltration with macrophages (brown) in mice infected with (**G**) and without (**H**) *H. suis*. The infiltration is more severe during *H. suis* infection. Original magnification: ×400. **I**, **J** H+/K+ATPase-staining showing parietal cells (brown) in mice infected with (**I**) and without (**J**) *H. suis*. The number of parietal cells is lower during *H. suis* infection. *H. suis* bacteria can be seen in the lumen of the gastric glands (arrow). Original magnification: ×400.

The *H. suis*-positive control group 4 showed significantly higher numbers of infiltrating B-cells and macrophages compared to the *H. suis*-negative control group 1 (*P* = 0.016 and 0.003, resp.) (Figure 2). Furthermore, a trend towards a higher number of infiltrating T-cells was seen in all *H. suis* infected groups 4–6 compared to the non-infected groups 1–3, although this was not significant (Figures 1 and 2).

Compared to the *H. suis*-positive control group 4, a lower number of infiltrating B-cells and macrophages was demonstrated in the 32 ppm bambermycin supplemented, *H. suis* infected group 5 (*P* = 0.068 and 0.001, resp.) as well as in the 64 ppm bambermycin supplemented, *H. suis* infected group 6 (*P* = 0.003 and 0.005, resp.) (Figure 2). In comparison to both groups that did not receive bambermycin (groups 1 and 4), all bambermycin-supplemented diet groups showed significantly lower scores for infiltration with inflammatory cells as well as infiltrating T-cells (*P* < 0.005 and < 0.001, resp.) (Figures 1 and 2). No significant differences were detected in the formation of lymphoid follicles between the groups.

The infiltration with inflammatory cells was positively correlated with the number of infiltrating T-cells and macrophages, while the number of infiltrating T-cells was positively correlated with the number of infiltrating B-cells and macrophages (Additional file 4). No correlations were found between the number of inflammatory cells and the number of colonizing *H. suis* bacteria per mg gastric tissue.

Positive correlations were found between the number of infiltrating macrophages and the expression of IL-8Kc, IL-8Li, IL-10, IL-17, IFN-γ, TNF-α and IL-1β; between the number of infiltrating T-cells and the expression of IL-8Li, IL-10 and IL-1β and between the number of infiltrating B-cells and the expression of IL-4, IL-10 and IL-12 (Additional file 4).

#### Gastric epithelial cell proliferation and death

An overview of the defined cell populations of each group is shown in Figure 1.

The *H. suis*-positive control group 4 showed significantly lower numbers of parietal cells compared to the *H. suis*-negative control group 1 (*P* = 0.032) (Figures 1 and 2). The 64 ppm bambermycin supplemented, *H. suis*-infected group 6, however, showed significantly higher numbers of parietal cells compared to the *H. suis*-positive control group 4 (*P* = 0.038). This was also the case for the 32 ppm bambermycin supplemented, *H. suis* infected group 5, although not significant. The number of apoptotic cells and epithelial cell proliferation did not differ between the groups.

Negative correlations were detected between the number of parietal cells and the number of infiltrating T-cells, B-cells, macrophages and inflammatory cells in general (Additional file 4).

#### Effect of bambermycin supplementation on the expression of markers for inflammation

**Figure 3:**
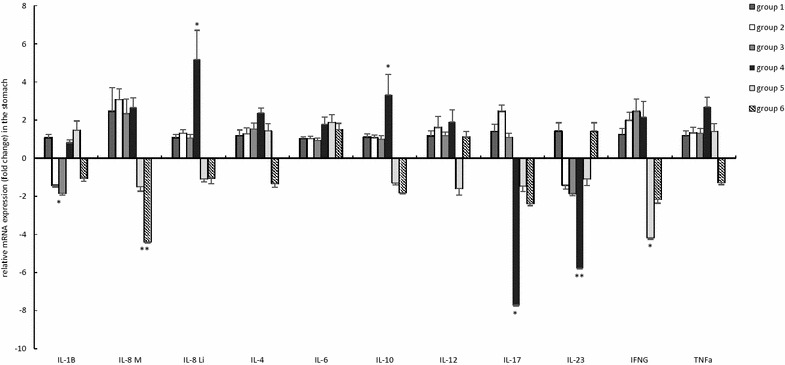
**General overview of relative fold changes of altered markers for inflammation in the bambermycin-supplemented and non-supplemented groups.** The data are presented as fold changes in gene expression normalized to 3 reference genes and relative to control groups 1 and 4 (i.e. group 2–4 relative to group 1 and group 5–6 relative to group 4) which are considered as 1. The fold changes are shown as means with the standard error of the mean. Statistical differences were calculated using the non-parametric Kruskal–Wallis H test SPSS statistics 24^®^. *Significant differences with the control group (*P* < 0.05), **significant differences with the control group (*P* < 0.005). Group 1 = *H. suis*-negative control group without bambermycin supplementation; group 2 = 32 ppm bambermycin supplemented, non-*H. suis* infected group; group 3 = 64 ppm bambermycin supplemented, non-*H. suis* infected group; group 4 = *H. suis*-positive control group without bambermycin supplementation; group 5 = 32 ppm bambermycin supplemented, *H. suis* infected group; group 6 = 64 ppm bambermycin supplemented, *H. suis* infected group.

The fold changes of altered markers for inflammation are represented in Figure 3 and Additional file 5.

The *H. suis*-positive control group 4 showed upregulated expressions of IL-4, IL-6, IL-8M, IL-8Li, IL-10, and TNF-α compared to the *H. suis*-negative control group 1 (*P* = 0.091, 0.115, 0.298, 0.002, 0.012 and 0.065, resp.). In addition, IL-17 and IL-23 were significantly downregulated (*P* < 0.001) (Additional file 5, Figure 3). Both bambermycin supplemented, *H. suis* infected groups 5 and 6 showed upregulated expressions of IL-4, IL-6 and IL-8Li in comparison with the *H. suis*-negative control group 1 (fold change = 3.24 ± 0.89, 2.95 ± 0.64 and 3.25 ± 0.54; *P* = 0.118, 0.066 and 0.002, resp. for group 5; fold change = 1.71 ± 0.48, 2.38 ± 0.51 and 3.44 ± 1.03; *P* = 0.970, 0.066 and 0.015, resp. for group 6), while IL-17 and IL-23 transcript levels were downregulated (fold change = 0.05 ± 0.02 and 0.15 ± 0.05; *P* < 0.001 and < 0.001, resp. for group 5; fold change = 0.03 ± 0.01 and 0.22 ± 0.07; *P* < 0.001 and = 0.001, resp. for group 6). The expression of TNF-α, however, was not altered during bambermycin supplementation in the *H. suis* infected groups 5 and 6 in comparison with the *H. suis*-negative control group 1. Since positive correlations were found between both, the altered fold changes of IL-4, IL-8Li, IL-17 and IL-23 were more pronounced in mice with a higher number of colonizing *H. suis* bacteria per mg gastric tissue (Additional file 4).

Compared to the *H. suis*-positive control group 4, the mRNA expressions of IL-8M and IFN-γ were downregulated in the 32 ppm bambermycin supplemented, *H. suis* infected group 5 (*P* = 0.100 and 0.015, resp.), as well as the IL-8M, IL-10 and IFN-γ transcript levels in the 64 ppm bambermycin supplemented, *H. suis* infected group 6 (*P* = 0.001, 0.077, 0.253, resp.).

In both bambermycin supplemented, *H. suis*-negative groups 2 and 3, the expression of IL-1β was significantly downregulated compared to the *H. suis*-negative control group 1 (*P* = 0.028 and 0.015, resp.). A similar observation was found for the 64 ppm bambermycin supplemented, *H. suis*-positive group compared to the *H. suis*-negative control group (fold change = 0.72 ± 0.13; *P* = 0.065).

#### Effect of bambermycin supplementation on the expression of markers for gastric acid secretion

**Figure 4:**
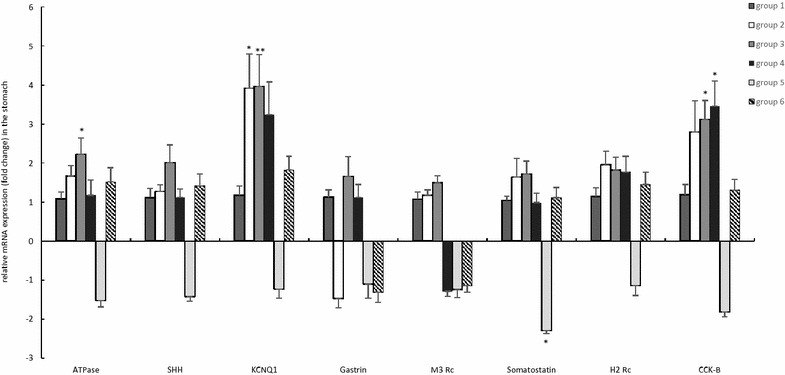
**General overview of relative fold changes of altered markers for gastric acid secretion in the bambermycin-supplemented and non-supplemented groups.** The data are presented as fold changes in gene expression normalized to 3 reference genes and relative to control groups 1 and 4 (i.e. group 2–4 relative to group 1 and group 5–6 relative to group 4) which are considered as 1. The fold changes are shown as means with the standard error of the mean. Statistical differences were calculated using the non-parametric Kruskal–Wallis H test SPSS statistics 24^®^. *Significant differences with the control group (*P* < 0.05), **significant differences with the control group (*P* < 0.005). Group 1 = *H. suis*-negative control group without bambermycin supplementation; group 2 = 32 ppm bambermycin supplemented, non-*H. suis* infected group; group 3 = 64 ppm bambermycin supplemented, non-*H. suis* infected group; group 4 = *H. suis*-positive control group without bambermycin supplementation; group 5 = 32 ppm bambermycin supplemented, *H. suis* infected group; group 6 = 64 ppm bambermycin supplemented, *H. suis* infected group.

The fold changes of altered markers for gastric acid secretion are presented in Figure 4 and Additional file 6.

In summary, the *H. suis*-positive control group 4 showed upregulated expressions of KCNQ1 and the CCK-B receptor in comparison with the *H. suis*-negative control group 1 (*P* = 0.119 and 0.032, resp.). In addition, the bambermycin supplemented groups 2–3 and 5 showed upregulated expressions of H+/K ATPase, Sonic Hedgehog, KCNQ1, M3 receptor and/or CCK-B receptor in comparison with the *H. suis*-negative and/or positive control groups 1 and 4 (Figure 4, Additional file 6).

Positive correlations were found between the altered expressions of KCNQ1, CCK-B receptor and somatostatin and the expressions of IL-8 and IL-10 (Additional file 4).

### Effect of bambermycin supplementation and/or *H. suis* infection on the gastric microbiota composition in mice

Despite several attempts, including altering the number of pyrosequencing cycles, no sequencing reads could be obtained from three mice of the *H. suis*-negative control group 1; 5 of group 2; 4 of group 3; 4 of group 4; 4 of group 5 and 3 of group 6. The other 25 samples yielded sufficient reads after pyrosequencing and were selected for further analysis. Pyrosequencing yielded between 400 and 4000 reads per sample (Additional file 7). A total of 70 118 final reads were attributed to 1290 species level OTUs for the 25 samples. Chimeric sequences represented 20% of the total sequencing reads and were thus excluded from the analysis.

**Figure 5:**
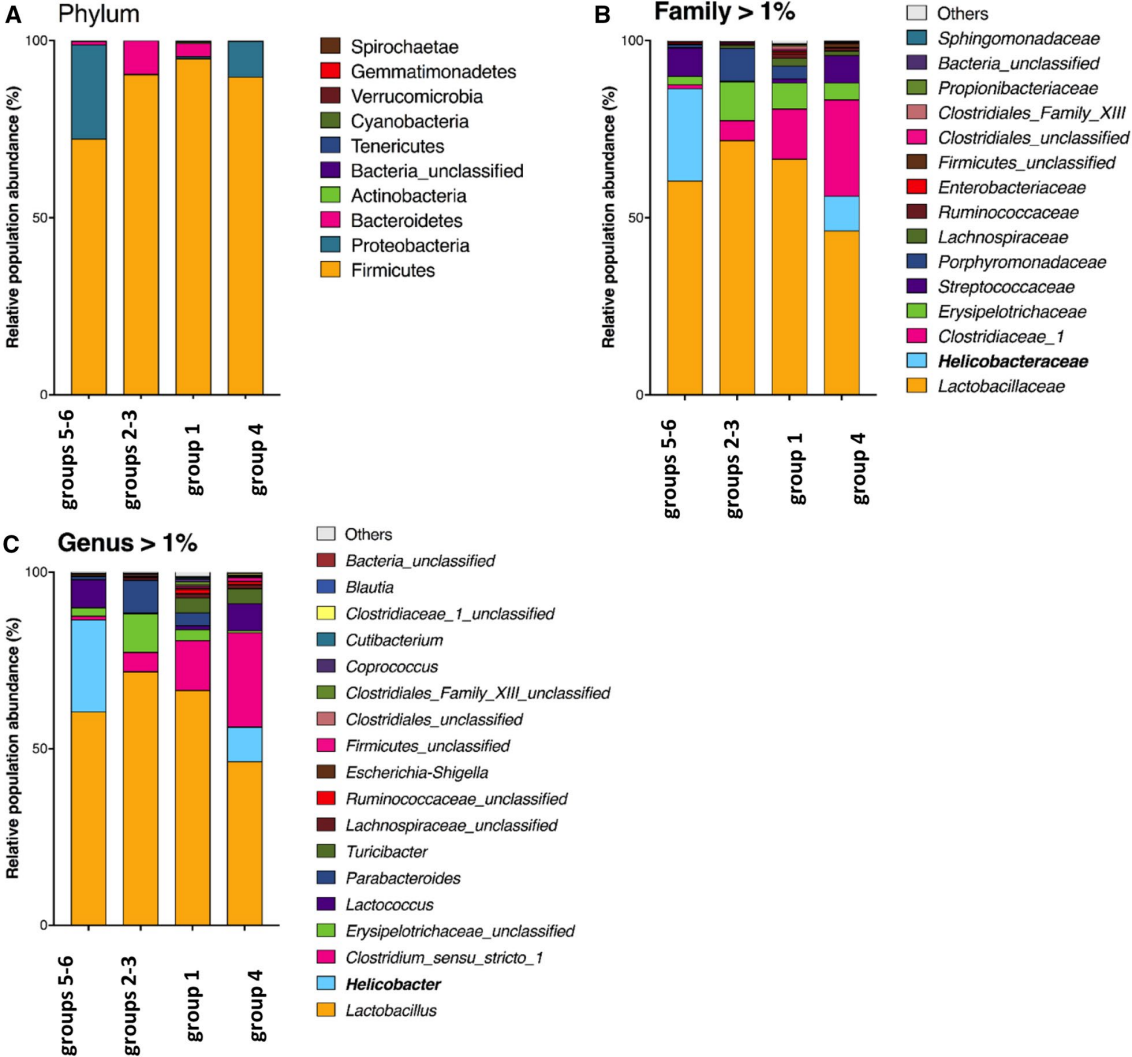
**Average gastric bacterial community compositions present in the bambermycin-supplemented and non-supplemented groups.** The cumulated histograms show the relative abundance of the identified taxa at phylum (**A**), family (**B**) or genus (**C**) level. At family and genus level, taxa with a relative abundance < 1% are merged in the category “others”. Group 1 = *H. suis*-negative control group without bambermycin supplementation; group 2 = 32 ppm bambermycin supplemented, non-*H. suis* infected group; group 3 = 64 ppm bambermycin supplemented, non-*H. suis* infected group; group 4 = *H. suis*-positive control group without bambermycin supplementation; group 5 = 32 ppm bambermycin supplemented, *H. suis* infected group; group 6 = 64 ppm bambermycin supplemented, *H. suis* infected group. The unclassified populations correspond to defined groups of the genus level for which a taxonomical classification assignation to the genus cannot be attributed. These populations are therefore labelled with the first defined superior hierarchical taxonomic level followed by “_unclassified” to prevent confusion.

In general, total bacterial community analysis showed that the most dominant phylum in the murine stomach was Firmicutes contributing up to 92%, followed by Bacteroides (4%) and Proteobacteria (4%). The relative abundance of other phyla was below 0.1%. On the family level, following populations (i.e. > 0.5%) were dominant in the murine stomach: Lactobacillaceae (63%), Clostridiaceae (9%), Erysipelotrichaceae (6%), Streptococcaceae (4%) and Porphyromonadaceae (4%). The major genera (i.e. > 0.5%) were *Lactobacillus* (63%), *Clostridium* sensu stricto (9%), unclassified *Erysipelotrichaceae* (5%), *Lactococcus* (4%), *Parabacteroides* (4%) and *Turicibacter* (2%). In two bambermycin supplemented, *H. suis*-positive mice (i.e. one of the 32 ppm supplemented group 5 and one of the 64 ppm supplemented group 6), however, Proteobacteria represented the major phylum (97%). Helicobacteriaceae (11%) and *Helicobacter* (11%) also represented dominant populations in the murine stomach, but only in the *H. suis*-positive groups 4–6. The average gastric bacterial community composition at the phylum, family and genus level present in the different groups is represented in Figures 5A–C, while the bacterial community composition of each individual mice is shown in Additional files 8 A–C.

Bambermycin supplementation and/or infection with *H. suis* had no effect on microbial diversity, richness and population evenness (Additional files 9 A–C). Furthermore, Unifrac weighted analysis as well as AMOVA and HOMOVA did not reveal significant differences regarding community structure and composition of the groups. Population structure and community membership, as determined by Bray–Curtis dissimilarity index, was also not different between the groups.

Phenotypic analysis of the murine gastric microbiota in general revealed the presence of 12 metabolic phenotypes of which ammonia oxidizer, dehalogenation, chitin degradation, xylan degrader, sulfide oxidizer and dinitrogen-fixing were the most abundant, each accounting for 78, 78, 14, 7, 7 and 6% of the gastric bacterial community, respectively. In the gastric microbiota of all mice, non-sporulating and Gram-positive bacteria were more abundant than sporulating and Gram-negative bacteria, respectively (70% vs 10% and 72% vs 0.5%, respectively). Presence of *H. suis* infection did not influence the phenotypic features of the gastric bacterial community. The bambermycin supplemented groups, however, showed significant lower abundance of sporulating and chitin degrading bacteria compared to the groups without bambermycin supplementation (3.0% vs 19.7%, *P* = 0.027 and 7.9% vs 23.7%, *P* = 0.027, respectively).

The taxa Helicobacteriaceae, *Helicobacter* and *H. suis* at family, genus and species level, respectively, were only present in the *H. suis* infected groups (11%, *P* < 0.05). Infection with *H. suis* did not influence the relative abundance of other taxa at phylum, family or genus level. At species level, *Christensenella* sp. EF603775 was only present in the *H. suis*–negative mice (0.041%), although this difference was not significant (*P* = 0.223). In general, mice that received bambermycin (i.e. groups 2, 3, 5 and 6) showed a relative lower abundance of the following taxa:

- At phylum family, genus and species level: *Firmicutes*_unclassified.
- At phylum, family and genus level: *Bacteria*_unclassified.
- At family level: Clostridiaceae_1.
- At genus level: Clostridiaceae_1_unclassified, *Coprococcus*, *Turicibacter*, *Clostridium*_sensu_stricto_1.
- At species level: *Coprococcus*_EF099198, *Coprococcus*_16S_OTU119, *Clostridiales*_Family_XIII_AB702776, *Clostridiales*_Family_XIII_16S_OTU162, *Clostridiales*_Family_XIII_EF604613, *Clostridiaceae*_1_16S_OTU75, *Clostridiaceae*_1_16S_OTU107, *Firmicutes*_16S_OTU195, *Firmicutes*_16S_OTU37, *Firmicutes*_16S_OTU43, *Firmicutes*_16S_OTU594, *Turicibacter*_EF406660, *Turicibacter*_DQ015666 and *Turicibacter*_EF406615 (Additional file 10).

The relative abundance of these taxa did not differ between the groups receiving different doses of in-feed bambermycin medication.

Interestingly, the relative abundance of these taxa was positively correlated with infiltration with inflammatory cells, T-cells, B-cells and macrophages as well as the expression of IFN-γ, gastrin and somatostatin, while negatively correlated with the number of parietal cells and the expression of H2 receptor, CCK-B receptor, H+/K+ATPase and KCNQ1 (Additional file 11).

## Discussion

In the present study, an infection with *H. suis* elicited increased infiltration with inflammatory cells such as B-cells, macrophages and T-cells. These findings are in line with previous studies where *H. suis* infection was associated with development of severe gastritis in experimentally and naturally infected pigs and experimentally infected mice and Mongolian gerbils [17, 34, 35]. Since macrophages produce several factors, such as IL-10, which provoke a Th2 response [36] and since B-cells promote the expansion of Th2 cells [37], infiltration with these inflammatory cells may have contributed to the observed Th2 response in *H. suis* infected mice.

The expression of IL-17, however, was downregulated in the *H. suis* infected mice, in contrast with other studies where a clear IL-17 upregulation and a mixed Th2/Th17 response was present in *H. suis* infected mice [17, 26, 34, 38, 39]. This might be caused by the absence of an IL-23 upregulation, a cytokine stimulating Th17 cell expansion [40]. As Flahou et al. showed an inverse correlation between the *H. suis* colonization rate and the expression of IL-17 [26], the observed high number of colonizing *H. suis* bacteria per mg gastric tissue might have contributed to the downregulation of IL-17. Still, it is not clear why a Th17 response was absent in this study.

BALB/c mice experimentally infected with *H. pylori* and C57BL/6 mice experimentally infected with *H. felis*, showed a decreased number of *Lactobacillus* spp. and an increased number of *Clostridium* spp., *Bacteroides* spp., *Prevotella* spp., *Eubacterium* spp., *Ruminococcus* spp., *Streptococcus* spp. and *E. coli* in their stomach [41, 42]. In another study, however, *H. pylori* infected C57BL/6 mice did not show gastric microbiota alterations [43]. In the present study, *H. suis* infection was not associated with a shift in the gastric microbiota. Often, the used mouse substrain is not mentioned and although the same strain can be used in different studies, the substrain may be different. These differences in genetic background may contribute to discrepancies between studies [44]. Differences in virulence between *Helicobacter* sp., stage of infection, and/or influence of the host immune response may also contribute to the discrepancies [45]. In future studies, it would be interesting to investigate a more long term effect of a *H. suis* infection on the gastric microbiota composition.

Since *H. suis* only grows in a biphasic medium with an acidic pH, standard antimicrobial susceptibility assays cannot be used for MIC determinations. The use of the combined agar and broth dilution method [20] may have influenced the results, as Butaye et al. demonstrated that medium composition has a clear impact on bambermycin activity [22]. For example, addition of sheep blood, hemoglobin, albumin, casein and starch as well as variations in pH and inoculum size affect the activity of bambermycin in vitro [10, 22]. Although for MIC determinations, a strict standardization of the test medium is preferable [22], no optimal medium exists to test bambermycin susceptibility of fastidious micro-organisms, such as *H. suis.* In comparison with the method described by Butaye et al. [22], the MIC endpoints of the reference strain *S. aureus* ATCC 29213 were 2, 3 and 4 times higher when using the broth microdilution procedure according to CLSI standards and the *H. suis* susceptibility assay conditions at pH 5 and 7, respectively. The presence of dextrose, casein and/or other components in Brucella broth may have contributed to the decreased activity of bambermycin.

To the best of our knowledge, no susceptibility data of bambermycin on *H. suis* has been published. In the present study, all *H. suis* strains showed a MIC-value of 8 μg/mL. Despite this relatively low MIC endpoint, bambermycin supplementation did not affect the *H. suis* colonization rate in vivo. As no specific clinical breakpoints for bambermycin against *H. suis* are available, prediction of the clinical efficacy based on in vitro testing solely is difficult. This is further complicated by the fact that, even in vitro, different components in the medium such as proteins, starch and lipid substances, may highly influence the antibacterial activity of bambermycin [22]. The gastric environment is far more complex than this in vitro environment, making it almost impossible to predict the activity of bambermycin in the stomach from results of MIC determinations.

Despite the absence of an effect on the *H. suis* colonization rate, parietal cell loss during *H. suis* infection was countered when bambermycin was supplemented. A decreased parietal cell mass has also been shown in *H. suis* infected BALB/c mice and Mongolian gerbils [17, 35]. It has been postulated that the loss of parietal cells might be due to a direct interaction of *H. suis* with these cells, since the bacterium can cause degenerative changes and necrosis of parietal cells in pigs, humans and rodent models [17, 46] and *H. suis* is able to directly interfere with cultured parietal cells, causing a significant impairment in cell viability [47]. Production of γ-glutamyl transpeptidase (GGT) by *H. suis* has been linked with these degenerative changes and/or impairment in cell viability [35]. It remains to be determined if bambermycin affects this direct interaction of the bacterium with parietal cells, without influencing its colonization capacity. The inverse correlation seen in the present and other studies [47], between severity of gastric inflammation and the number of parietal cells indicates that not only direct interaction of this bacterium with these host cells, but also inflammation may play a role in parietal cell death in *H. suis* infected hosts.

Several studies attribute a role to *H. suis* in the development of hyperkeratosis and ulceration of the non-glandular stratified squamous epithelium of the *Pars oesophagea* of the porcine stomach, although *H. suis* does not colonize this region [4]. It is not completely clear how exactly *H. suis* influences ulcer development, but a recent study indicates that alterations in gastric acid secretion may be involved [3]. Indeed, in *H. suis* infected 6–8 months old pigs with severe hyperkeratosis and erosions of the non-glandular part of the stomach, expression of markers for gastric acid secretion was downregulated. It was hypothesized that decreased gastric acid secretion may affect the composition of the *Pars oesophageal* microbiota. Indeed, compared to non-infected, 6–8 months old pigs with no obvious lesions, higher numbers of *Fusobacterium gastrosuis* [48], were detected in the *Pars oesophagea* of *H. suis* infected pigs with hyperkeratosis and erosions of the *Pars oesophagea* and downregulated markers for gastric acid secretion. In the present study, bambermycin supplementation resulted in an upregulation of several markers for gastric acid secretion and seemed to counter the parietal cell mass loss during *H. suis* infection. It remains to be determined if bambermycin affects gastric ulcer development in *H. suis* infected pigs.

Although an improved feed conversion and growth has been demonstrated in pigs, cattle and poultry during bambermycin supplementation, the effect on gastro-intestinal inflammation had not been investigated before [49–52]. Here, the increased infiltration with macrophages, T-cells and B-cells and upregulated expressions of IL-8M, IL-10, TNF-α and IFN-γ in *H. suis* infected mice was countered when bambermycin was supplemented in the diet, even though this antibiotic apparently did not influence *H. suis* colonization. Similarly, in the stomach of the non-*H. suis* infected mice treated with bambermycin, a decreased T-cell and macrophage infiltration was observed as well as a downregulated expression of the pro-inflammatory cytokine IL-1β, further indicating that bambermycin may alter the function of inflammatory cells resulting in a more tempered host immune response. The mechanism behind this anti-inflammatory effect is not clear. It has been hypothesized that macrolides, cyclines and streptogramins may accumulate in phagocytic cells, reducing the production of pro-inflammatory cytokines [53]. Macrolides may also inhibit T-cell maturation and proliferation [54]. It is not known if similar mechanisms are involved for bambermycin.

In the present study, bambermycin supplementation did not cause major shifts in *Lactobacillus* spp., which have been considered to exert a beneficial effect in the intestinal tract [10]. On the other hand, the presence of bacterial species belonging to *Firmicutes*, *Turicibacter*, *Coprococcus*, *Clostridiaceae*, *Clostridiales* family XIII and *Clostridium senso stricto* 1 taxa was positively correlated with infiltration of inflammatory cells as well as expression of markers for inflammation. This might indicate that some bacteria belonging to these taxa might exert unfavorable effects on the host. Since the relative abundance of these taxa was decreased during bambermycin supplementation, an altered gastric microbiota composition may also have contributed to the anti-inflammatory effect of bambermycin. Nevertheless, as most of these species are yet unknown, further research is necessary to confirm or deny this hypothesis. Finally, ammonia oxidizers accounted for 78% of the gastric bacterial community. These bacteria produce nitric oxide, which may exert damaging effects and contribute to gastritis. It has been shown that bambermycin exhibits an anti-oxidative effect by scavenging free nitric oxide radicals in vitro [55], which may also play a role in the observed anti-inflammatory effect of in-feed bambermycin medication.

In conclusion, bambermycin supplementation did not affect *H. suis* colonization, but did decrease gastric inflammation and inhibited the effects of a *H. suis* infection on parietal cell loss. Not only direct interaction of *H. suis* with parietal cells, but also inflammation may play a role in death of these gastric acid producing cells.

## Additional files

**Additional file 1.** **List of primers used in quantitative RT-PCR for gene expression analysis of markers for gastric acid secretion and inflammation.**

**Additional file 2.** **Number and colonization density of** ***H. suis*** **in groups 4-6.** (A) Number of *H. suis* bacteria per mg gastric tissue of group 4-6. Data are shown as log10 values of the average of number of *H. suis* bacteria per mg tissue with standard deviation. (B) Colonization density of *H. suis* in the stomach of group 4-6. Data are shown as the average of the colonization score for each group with standard deviation. Group 4 = *H. suis*-positive control without bambermycin supplementation; group 5 = 32 ppm bambermycin supplemented, *H. suis* infected group; group 6 = 64 ppm bambermycin supplemented, *H. suis* infected group.

**Additional file 3.** **Immunohistochemical** ***Helicobacter*** **(A)**, **caspase-3 (B) and KI-67 (C) staining of a mouse stomach, showing** ***H. suis*** **colonization, apoptotic cells and replicating cells, respectively.** (A) *H. suis* bacteria (brown) present in the glands of the antrum of a stomach of a *H. suis*-positive control mouse not treated with bambermycin. Original magnification: ×400. (B) Apoptotic cells (brown) present in a stomach of a *H. suis*-negative mouse treated with bambermycin. Original magnification: ×400. (C) Replicating cells (brown) present in a stomach of a *H. suis*-negative mouse treated with bambermycin. Original magnification: ×400.

**Additional file 4.** **Overview of important correlations.** r = Pearson correlation coefficient, calculated using SPSS Statistics 24^®^. A r-value close to 1 indicates a strong, positive correlation, whereas a r-value of -1 indicates a strong, negative correlation. *P*-values lower than 0.05 are considered to be significant.

**Additional file 5.** **Overview of the relative fold changes of altered markers for inflammation in the bambermycin-supplemented and non-supplemented groups.** The data are presented as fold changes in gene expression normalized to 3 reference genes and relative to control groups 1 and 4 (i.e. group 2-4 relative to group 1 and group 5-6 relative to group 4) which are considered as 1. The fold changes are shown as means with the standard error of the mean. Statistical differences were calculated using the non-parametric Kruskal–Wallis H test SPSS statistics 24^®^. A P-value lower than 0.05 is considered to be significant. Group 2 = 32 ppm bambermycin supplemented, non-*H. suis* infected group; group 3 = 64 ppm bambermycin supplemented, non-*H. suis* infected group; group 4 = *H. suis*-positive control group without bambermycin supplementation; group 5 = 32 ppm bambermycin supplemented, *H. suis* infected group; group 6 = 64 ppm bambermycin supplemented, *H. suis* infected group.

**Additional file 6.** **Overview of the relative fold changes of altered markers for gastric acid secretion in the bambermycin-supplemented and non-supplemented groups.** The data are presented as fold changes in gene expression normalized to 3 reference genes and relative to control groups 1 and 4 (i.e. group 2-4 relative to group 1 and group 5-6 relative to group 4) which are considered as 1. The fold changes are shown as means with the standard error of the mean. Statistical differences were calculated using the non-parametric Kruskal–Wallis H test SPSS statistics 24^®^. A *P*-value lower than 0.05 is considered to be significant. Group 2 = 32 ppm bambermycin supplemented, non-*H. suis* infected group; group 3 = 64 ppm bambermycin supplemented, non-*H. suis* infected group; group 4 = *H. suis*-positive control group without bambermycin supplementation; group 5 = 32 ppm bambermycin supplemented, *H. suis* infected group; group 6 = 64 ppm bambermycin supplemented, *H. suis* infected group.

**Additional file 7.** **Overview of the number of pyrosequencing reads for each mice.** Group 1 = *H. suis*-negative control group without bambermycin supplementation; group 2 = 32 ppm bambermycin supplemented, non-*H. suis* infected group; group 3 = 64 ppm bambermycin supplemented, non-*H. suis* infected group; group 4 = *H. suis*-positive control group without bambermycin supplementation; group 5 = 32 ppm bambermycin supplemented, *H. suis* infected group; group 6 = 64 ppm bambermycin supplemented, *H. suis* infected group.

**Additional file 8.** **Bacterial community compositions present in the stomach of each individual mice.** The cumulated histograms show the relative abundance of the identified taxa at phylum (A), family (B) or genus (C) level. At family and genus level, taxa with a relative abundance < 1% are merged in the category “others”. M1_ = group 1 = *H. suis*-negative control group without bambermycin supplementation; M2_ = group 2 = 32 ppm bambermycin supplemented, non-*H. suis* infected group; M3_ = group 3 = 64 ppm bambermycin supplemented, non-*H. suis* infected group; M4_ = group 4 = *H. suis*-positive control group without bambermycin supplementation; M5_ = group 5 = 32 ppm bambermycin supplemented, *H. suis* infected group; M6_ = group 6 = 64 ppm bambermycin supplemented, *H. suis* infected group. The unclassified populations correspond to defined groups of the genus level for which a taxonomical classification assignation to the genus cannot be attributed. These populations are therefore labelled with the first defined superior hierarchical taxonomic level followed by “_unclassified” to prevent confusion.

**Additional file 9.** **Overview of the gastric bacterial richness, diversity and evenness of the bambermycin-supplemented and non-supplemented groups.** Gastric bacterial richness (A), diversity (B) and evenness (C). The data are represented as box plots: the bottom and top of the box represent the first and the third quartile, the line in the box represents the median and the whiskers represent the minimum and maximum values. Group 1 = *H. suis*-negative control group without bambermycin supplementation; group 2 = 32 ppm bambermycin supplemented, non-*H. suis* infected group; group 3 = 64 ppm bambermycin supplemented, non-*H. suis* infected group; group 4 = *H. suis*-positive control group without bambermycin supplementation; group 5 = 32 ppm bambermycin supplemented, *H. suis* infected group; group 6 = 64 ppm bambermycin supplemented, *H. suis* infected group.

**Additional file 10.** **An overview of the main differences in relative abundance of taxa at phylum, family, genus and species level in the bambermycin-supplemented and non-supplemented groups.** The data are presented as the mean relative abundance of the taxa with the standard error of the mean. Statistical differences were calculated using the non-parametric Kruskal–Wallis tests with Tukey post hoc tests and Benjamini–Hochberg False Discovery Rate were performed using STAMP^®^. A *P*-value lower than 0.05 is considered to be significant.

**Additional file 11.** **Overview of important correlations between the gastric bacterial community and the number of inflammatory cells, parietal cells and expression of markers for inflammation and gastric acid secretion.** r = Pearson correlation coefficient, calculated using SPSS Statistics 24^®^. A r-value close to 1 indicates a strong, positive correlation, whereas a r-value of -1 indicates a strong, negative correlation. *P*-values lower than 0.05 are considered to be significant.
