# Supplementary material for: In-feed bambermycin medication induces anti-inflammatory effects and prevents parietal cell loss without influencing Helicobacter suis colonization in the stomach of mice
Source: Vet Res. 2018 Apr 10;49:35. doi: 10.1186/s13567-018-0530-1 (PMC5894178; doi:10.1186/s13567-018-0530-1)
Supplement: Supplementary file 1 — Additional file 1. List of primers used in quantitative RT-PCR for gene expression analysis of markers for gastric acid secretion and inflammation. [file 13567_2018_530_MOESM1_ESM.doc]

**Additional file 1**: List of primers used in quantitative RT-PCR for gene expression analysis of markers for gastric acid secretion and inflammation.

| **Primer** | **Sequence (5’-3’)** | **Reference** |
| --- | --- | --- |
| Sonic Hedgehog-forward | AGCAGGTTTCGACTGGGTCT | [1] |
| Sonic Hedgehog-reverse | GCCACGGAGTTCTCTGCTT | [1] |
| Somatostatin-forward | GTCCTGGCTTTGGGCGGTGTCA | [2] |
| Somatostatin-reverse | TGCAGCTCCAGCCTCATCTCGT | [2] |
| Histamine 2 receptor-forward | GCCACCATCAGAGAACACAAAG | Adjusted from [3] |
| Histamine 2 receptor-reverse | AAGGAAACCAGCAGACGATGAA | Adjusted from [3] |
| Muscarinic 3 receptor-forward | ACCAAGCTACCCTCCTCAGA | Adjusted from [4] |
| Muscarinic 3 receptor-reverse | GACAGTTGTCACGGTCATCC | [4] |
| Gastrin-forward | AGCGCCAGTTCAACAAGCT | [5] |
| Gastrin-reverse | CCAAAGTCCATCCATCCGTAG | [5] |
| Cholecystokinin B receptor-forward | CTGGCTGTCGCTTGCATGCC | Adjusted from [6] |
| Cholecystokinin B receptor-reverse | GCAGTGGTCGGCAGATGGCG | Adjusted from [6] |
| H+/K+ APTase-forward | AGATGTCCTCATCCGCAAGACAC | [1] |
| H+/K+ APTase-reverse | CAGCCAATGCAGACCTGGAA | [1] |
| KCNQ1-forward | AACAGAATTGTCAAGTTCCTC | [7] |
| KCNQ1-reverse | AGACTGAAGGTGCCATTG | [7] |
| H2afz-forward | GGTATCACCCCTCGTCACTT | [8] |
| H2afz-reseverse | TCAGCGATTTGTGGATGTGT | [8] |
| HPRT-forward | CAGGCCAGACTTTGTTGGAT | [8] |
| HPRT-reverse | TTGCGCTCATCTTAGGCTTT | [8] |
| PPIa-forward | AGCATACAGGTCCTGGCATC | [8] |
| PPIa-reverse | TTCACCTTCCCAAAGACCAC | [8] |
| IL1β-forward | CACCTCACAAGCAGAGCACAAG | [9] |
| IL1β-reverse | GCATTAGAAACAGTCCAGCCCATAC | [9] |
| IL8KC-forward | GCTGGGATTCACCTCAAGAA | [10] |
| IL8KC-reverse | TCTCCGTTACTTGGGGACAC | [10] |
| IL8MIP-forward | TGCCTGAAGACCCTGCCAAGG | [10] |
| IL8MIP-reverse | GTTAGCCTTGCCTTTGTTCAG | [10] |
| IL8Lix-forward | CTCAGTCATAGCCGCAACCGAGC | [10] |
| IL8Lix-reverse | CCGTTCTTTCCACTGCGAGTGC | [10] |
| IL4-forward | GGTCTCAACCCCCAGCTAGT | [11] |
| IL4-reverse | GCCGATGATCTCTCTCAAGTGAT | [11] |
| IL6-forward | TAGTCCTTCCTACCCCAATTTCC | [11] |
| IL6-reverse | TTGGTCCTTAGCCACTCCTTC | [11] |
| IL10-forward | CTGGACAACATACTGCTAACCG | [12] |
| IL10-reverse | GGGCATCACTTCTACCAGGTAA | [12] |
| IL12-forward | ACCTGTGACACGCCTGAAGAAG | [13] |
| IL12-reverse | TGTGGAGCAGCAGATGTGAGTG | [13] |
| IL17-forward | CAGGACGCGCAAACATGA | [14] |
| IL17-reverse | GCAACAGCATCAGAGACACAGAT | [14] |
| IL23-forward | CACCTCCCTACTAGGACTCAGC | [15] |
| IL23-reverse | TGGGCATCTGTTGGGTCT | [15] |
| IFNγ-forward | CTGACCTAGAGAAGACACAT | [16] |
| IFNγ-reverse | GGTCAGTGAAGTAAAGGTAC | [16] |
| TNFα-forward | ACCCTGGTATGAGCCCATATAC | [10] |
| TNFα-reverse | ACACCCATTCCCTTCACAGAG | [10] |

[1] Fukuhara S, Matsuzaki J, Tsugawa H, Masaoka T, Miyoshi S, Mori H, Fukushima Y, Yasui M, Kanai T, Suzuki H (2014) Mucosal expression of aquaporin-4 in the stomach of histamine type 2 receptor knockout mice and *Helicobacter pylori*-infected mice. J Gastroenterol Hepatol 29:53–59.

[2] Takaishi S, Wang TC (2007) Gene expression profiling in a mouse model of *Helicobacter*-induced gastric cancer. Cancer Sci 98:284–293.

[3] Osawa H, Kita H, Ohnishi H, Mutoh H, Ishino Y, Satoh K, Sugano K (2005) Histamine-2 receptor expression in gastric mucosa before and after *Helicobacter pylori* cure. Aliment Pharmacol Ther 21 Suppl 2:92–98.

[4] Kitazawa T, Asakawa K, Nakamura T, Teraoka H, Unno T, Komori S, Yamada M (2009) M3 muscarinic receptors mediate positive inotropic responses in mouse atria: a study with muscarinic receptor knockout mice. 330:487–493.

[5] Du GM, Liu MJ, Parvizi N, Zhao RQ (2013) Ectopic expression of ghrelin affects gastric H+-K+-ATPase activity and expression of GHR/IGF-1 system in weaned mice. Regul Pept 186:12–17.

[6] Zhang G, Ducatelle R, Mihi B, Smet A, Flahou B, Haesebrouck F (2016) *Helicobacter suis* affects the health and function of porcine gastric parietal cells. Vet Res 47:101.

[7] Jain RN, Brunkan CS, Chew CS, Samuelson LC (2006) Gene expression profiling of gastrin target genes in parietal cells. Physiol Genomics 24:124–132.

[8] Flahou B, Haesebrouck F, Pasmans F, D’Herde K, Driessen A, van Deun K, Smet A, Duchateau L, Chiers K, Ducatelle R (2010) *Helicobacter suis* causes severe gastric pathology in mouse and Mongolian gerbil models of human gastric disease. PLoS One 5:1–11.

[9] Catrysse L, Farhang Ghahremani M, Vereecke L, Youssef SA, Mc Guire C, Sze M, Weber A, Heikenwalder M, de Bruin A, Beyaert R, van Loo G (2016) A20 prevents chronic liver inflammation and cancer by protecting hepatocytes from death. Cell Death Dis 7:e2250.

[10] Liu C, Rossi M, Lindén S, Padra M, Blaecher C, Bauwens E, Joosten M, Flahou B, Van den Broeck W, Ducatelle R, Haesebrouck F, Smet A (2016) The *Helicobacter heilmannii* hofE and hofF genes are essential for colonization of the gastric mucosa and play a role in IL-1beta-induced gastric MUC13 Expression. Helicobacter 21:504-522.

[11] Castiglioni A, Corna G, Rigamonti E, Basso V, Vezzoli M, Monno A, Almada AE, Mondino A, Wagers AJ, Manfredi AA, Rovere-Querini P (2015) FOXP3+ T cells recruited to sites of sterile skeletal muscle injury regulate the fate of satellite cells and guide effective tissue regeneration. PLoS One 10:1–18.

[12] Huang ZH, Reardon CA, Getz GS, Maeda N, Mazzone T (2015) Selective suppression of adipose tissue apoE expression impacts systemic metabolic phenotype and adipose tissue inflammation. J Lipid Res 56:215–26.

[13] Ichikawa S, Miyake M, Fujii R, Konishi Y (2012) MyD88 associated ROS generation is crucial for *Lactobacillus* induced IL-12 production in macrophage. PLoS One 7:e35880.

[14] Lee K, Won HY, Bae MA, Hong J-H, Hwang ES (2011) Spontaneous and aging-dependent development of arthritis in NADPH oxidase 2 deficiency through altered differentiation of CD11b+ and Th/Treg cells. Proc Natl Acad Sci U S A 108:9548–9553.

[15] Tortola L, Rosenwald E, Abel B, Blumberg H, Schäfer M, Coyle AJ, Renauld JC, Werner S, Kisielow J, Kopf M (2012) Psoriasiform dermatitis is driven by IL-36-mediated DC-keratinocyte crosstalk. J Clin Invest 122:3965–3976.

[16] Flahou B, Deun K Van, Pasmans F, Smet A, Volf J, Rychlik I, Ducatelle R, Haesebrouck F (2012) The local immune response of mice after *Helicobacter suis* infection: strain differences and distinction with *Helicobacter pylori*. Vet Res 43:1.
