# Supplementary material for: In-feed bambermycin medication induces anti-inflammatory effects and prevents parietal cell loss without influencing Helicobacter suis colonization in the stomach of mice
Source: Vet Res. 2018 Apr 10;49:35. doi: 10.1186/s13567-018-0530-1 (PMC5894178; doi:10.1186/s13567-018-0530-1)
Supplement: Supplementary file 2 — Additional file 2. Number and colonization density of H. suis in groups 4-6. (A) Number of H. suis bacteria per mg gastric tissue of group 4-6. Data are shown as log10 values of the average of number of H. suis bacteria per mg tissue with standard deviation. (B) Colonization density of H. suis in the stomach of group 4-6. Data are shown as the average of the colonization score for each group with standard deviation. Group 4 = H. suis-positive control without bambermycin supplementation; group 5 = 32 ppm bambermycin supplemented, H. suis infected group; group 6 = 64 ppm bambermycin supplemented, H. suis infected group. [file 13567_2018_530_MOESM2_ESM.docx]

|  |  |
| --- | --- |

**B**

**A**
