# Supplementary figures and images for: In-feed bambermycin medication induces anti-inflammatory effects and prevents parietal cell loss without influencing Helicobacter suis colonization in the stomach of mice
Source: Vet Res. 2018 Apr 10;49:35. doi: 10.1186/s13567-018-0530-1 (PMC5894178; doi:10.1186/s13567-018-0530-1)

**B**

| **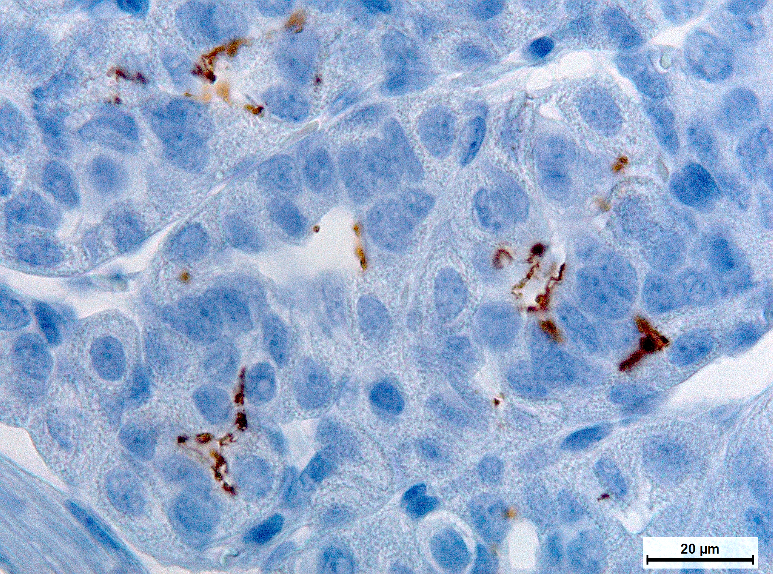**  **A** | **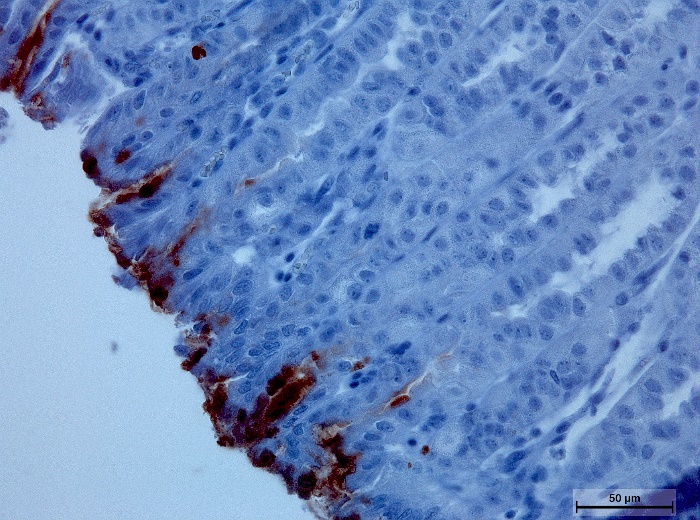** |
| --- | --- |
| **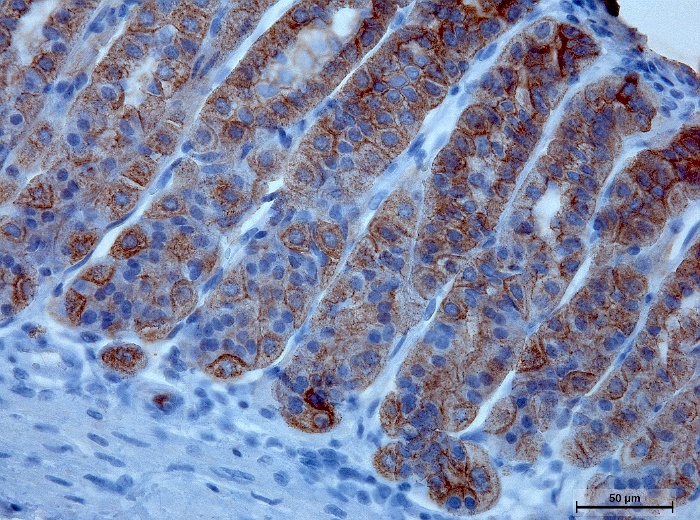**  **C** |  |

Supplement: Supplementary file 3 — Additional file 3. Immunohistochemical Helicobacter (A), caspase-3 (B) and KI-67 (C) staining of a mouse stomach, showing H. suis colonization, apoptotic cells and replicating cells, respectively. (A) H. suis bacteria (brown) present in the glands of the antrum of a stomach of a H. suis-positive control mouse not treated with bambermycin. Original magnification: ×400. (B) Apoptotic cells (brown) present in a stomach of a H. suis-negative mouse treated with bambermycin. Original magnification: ×400. (C) Replicating cells (brown) present in a stomach of a H. suis-negative mouse treated with bambermycin. Original magnification: ×400. [file 13567_2018_530_MOESM3_ESM.docx]

| 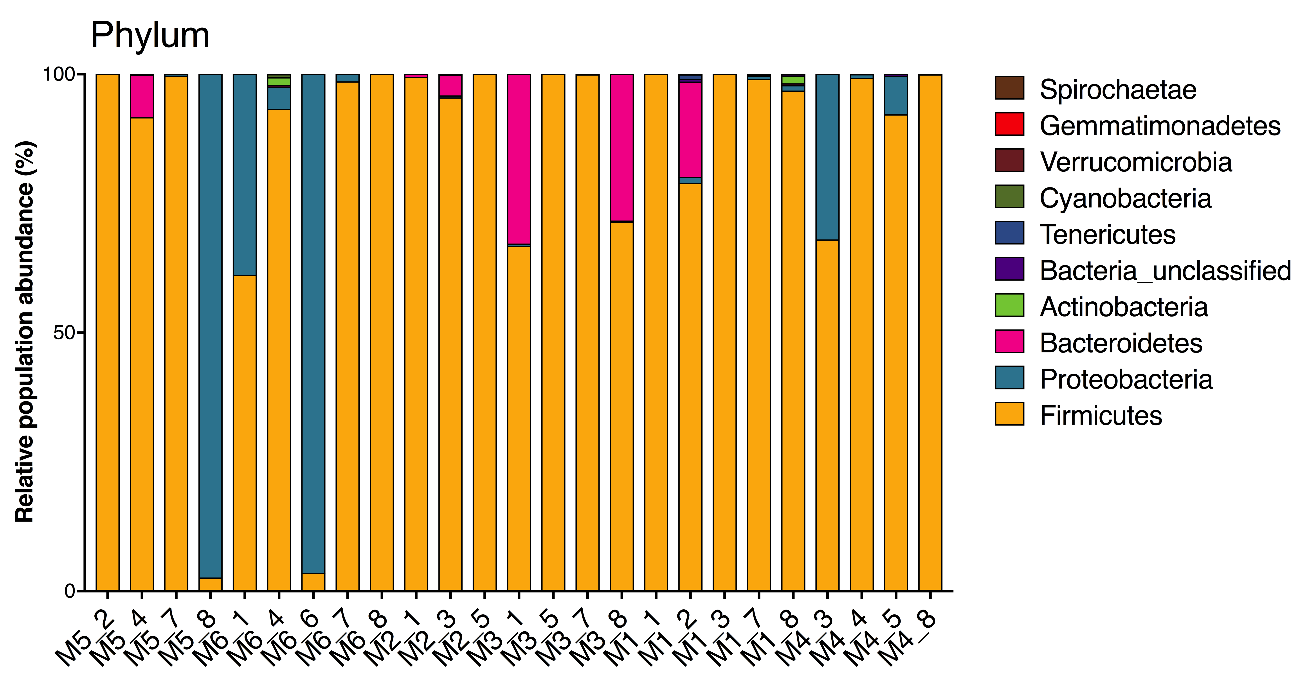  **A**  **B** |
| --- |
| 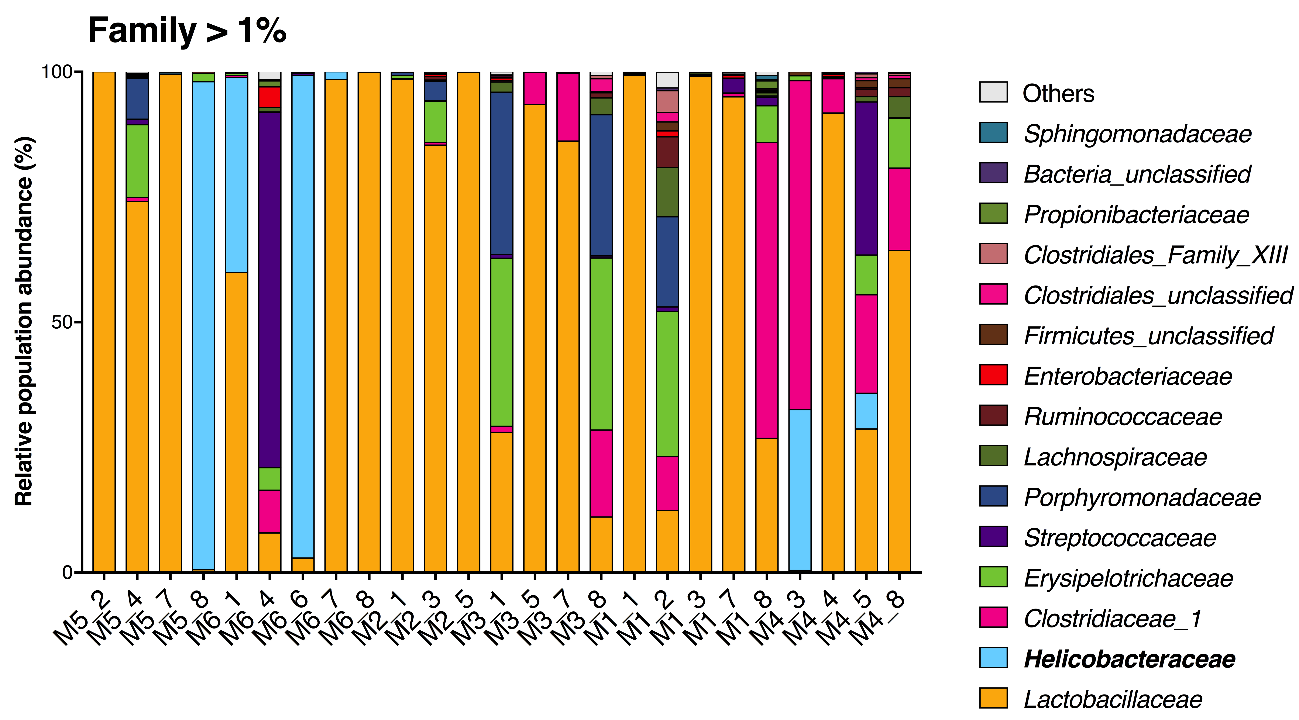 |
| 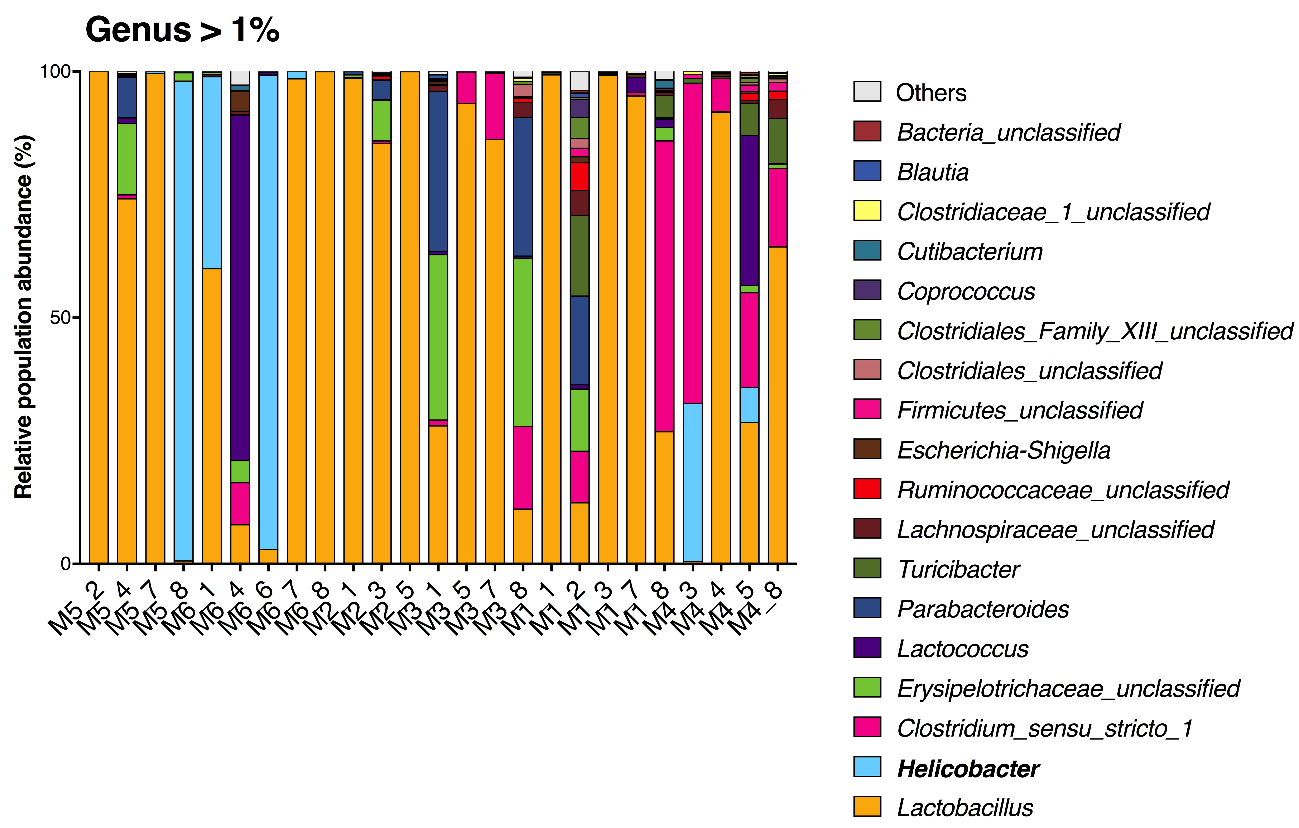 |

**C**

Supplement: Supplementary file 8 — Additional file 8. Bacterial community compositions present in the stomach of each individual mice. The cumulated histograms show the relative abundance of the identified taxa at phylum (A), family (B) or genus (C) level. At family and genus level, taxa with a relative abundance < 1% are merged in the category “others”. M1_ = group 1 = H. suis-negative control group without bambermycin supplementation; M2_ = group 2 = 32 ppm bambermycin supplemented, non-H. suis infected group; M3_ = group 3 = 64 ppm bambermycin supplemented, non-H. suis infected group; M4_ = group 4 = H. suis-positive control group without bambermycin supplementation; M5_ = group 5 = 32 ppm bambermycin supplemented, H. suis infected group; M6_ = group 6 = 64 ppm bambermycin supplemented, H. suis infected group. The unclassified populations correspond to defined groups of the genus level for which a taxonomical classification assignation to the genus cannot be attributed. These populations are therefore labelled with the first defined superior hierarchical taxonomic level followed by “_unclassified” to prevent confusion. [file 13567_2018_530_MOESM8_ESM.docx]

| **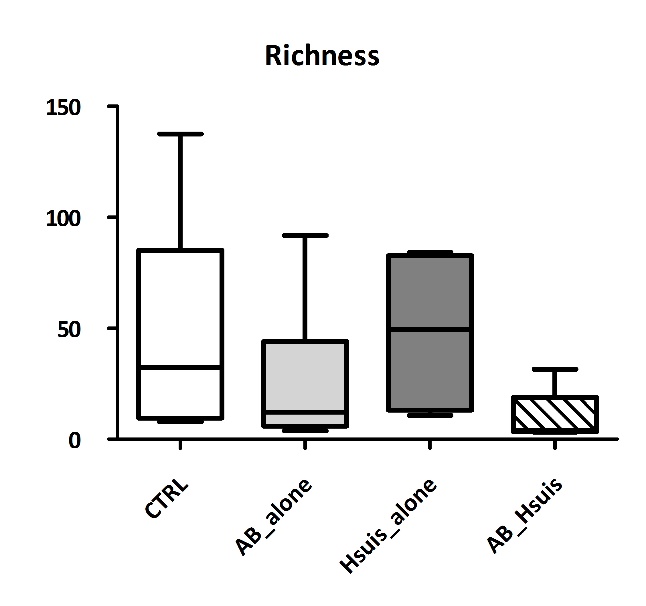**  **group 1**  **groups 2-3**  **group 4**  **groups 5-6** | 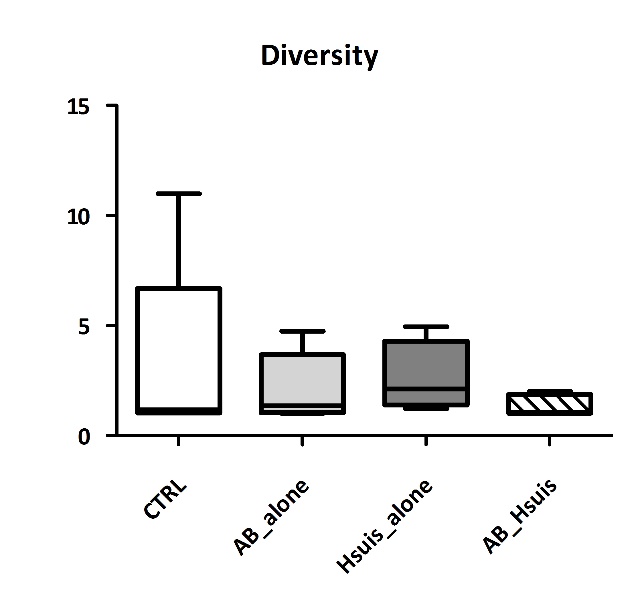  **group 1**  **groups 2-3**  **group 4**  **groups 5-6** | **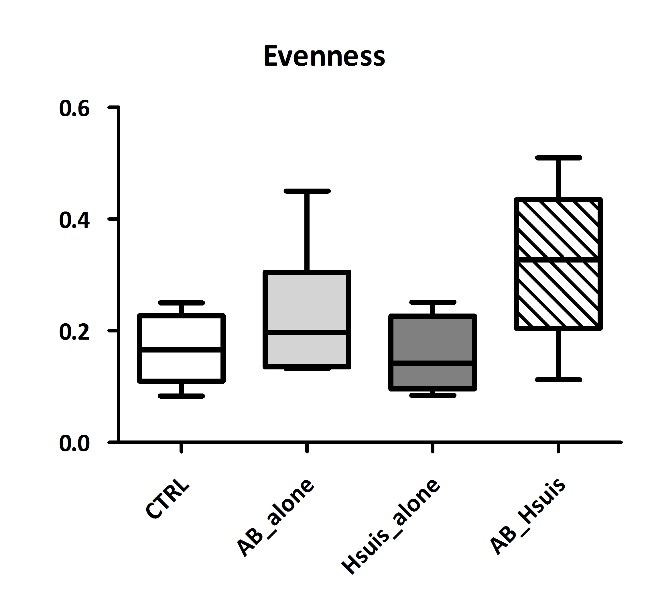**  **C**  **group 1**  **groups 2-3**  **group 4**  **groups 5-6** |
| --- | --- | --- |

**B**

**A**

Supplement: Supplementary file 9 — Additional file 9. Overview of the gastric bacterial richness, diversity and evenness of the bambermycin-supplemented and non-supplemented groups. Gastric bacterial richness (A), diversity (B) and evenness (C). The data are represented as box plots: the bottom and top of the box represent the first and the third quartile, the line in the box represents the median and the whiskers represent the minimum and maximum values. Group 1 = H. suis-negative control group without bambermycin supplementation; group 2 = 32 ppm bambermycin supplemented, non-H. suis infected group; group 3 = 64 ppm bambermycin supplemented, non-H. suis infected group; group 4 = H. suis-positive control group without bambermycin supplementation; group 5 = 32 ppm bambermycin supplemented, H. suis infected group; group 6 = 64 ppm bambermycin supplemented, H. suis infected group. [file 13567_2018_530_MOESM9_ESM.docx]
