# Supplementary material for: In-feed bambermycin medication induces anti-inflammatory effects and prevents parietal cell loss without influencing Helicobacter suis colonization in the stomach of mice
Source: Vet Res. 2018 Apr 10;49:35. doi: 10.1186/s13567-018-0530-1 (PMC5894178; doi:10.1186/s13567-018-0530-1)
Supplement: Supplementary file 4 — Additional file 4. Overview of important correlations. r = Pearson correlation coefficient, calculated using SPSS Statistics 24®. A r-value close to 1 indicates a strong, positive correlation, whereas a r-value of -1 indicates a strong, negative correlation. P-values lower than 0.05 are considered to be significant. [file 13567_2018_530_MOESM4_ESM.docx]

**Additional file 4 Overview of important correlations**

| **Number of cells** | **Number of infiltrating cells** |
| --- | --- |
| Infiltration with inflammatory cells | T-cells  r = 0.691  *P*-value < 0.001  Macrophages  r = 0.259  *P*-value = 0.076 |
| T-cells | B-cells  r = 0.326  *P-*value = 0.024  Macrophages  r = 0.543  *P-*value < 0.001 |
| Parietal cells | Infiltration with inflammatory cells  r = -0.222  *P-*value = 0.129  T-cells  r = -0.375  *P-*value = 0.009  B-cells  r = -0.278  *P-*value = 0.055  Macrophages  r = -0.264,  *P-*value = 0.070 |
| **Number of infiltrating cells** | **Expression of markers for inflammation** |
| T-cells | IL-8Li  r = 0.237  *P-*value = 0.117  IL-10  r = 0.323  *P-*value = 0.048  IL-1β  r = 0.228  *P-*value = 0.120 |
| B-cells | IL-4  r = 0.260  *P-*value = 0.085  IL-10  r = 0.554  *P-*value < 0.001  IL-12  r = 0.518  *P-*value < 0.001 |
| Macrophages | IL-8Kc  r = 0.389  *P-*value = 0.007  IL-8Li  r = 0.659  *P-*value < 0.001  IL-10  r = 0.401  *P-*value = 0.013  IL-17  r = 0.284  *P-*value = 0.053  IFN-γ  r = 0.413  *P-*value = 0.005  TNF-α  r = 0.248  *P-*value = 0.093  IL-1β  r = 0.293  *P-*value = 0.044 |
| **Gene expression of markers for inflammation** | **Number of *H. suis* bacteria** |
| IL-4 | r = 0.337  *P-*value = 0.024 |
| IL-6 | r = 0.517  *P-*value < 0.001 |
| IL-8Li | r = 0.274  *P-*value = 0.069 |
| IL-17 | r = -0.264  *P-*value = 0.073 |
| IL-23 | r = -0.201  *P-*value = 0.180 |
| **Gene expression of markers for gastric acid secretion** | **Gene expression of markers for inflammation** |
| KCNQ1 | IL-8Kc  r = 0.290  *P-*value = 0.050  IFN-γ  r = 0.300  *P-*value = 0.054 |
| CCK-B receptor | IL-8Kc  r = 0.381  *P-*value = 0.010  IL-8M  r = 0.257  *P-*value = 0.092  IL-8Li  r = 0.427  *P-*value = 0.004  IL-10  r = 0.252  *P-*value = 0.138  TNF-α  r = 0.214  *P-*value = 0.162 |
| Somatostatin | IL-8M  r = 0.413  *P-*value = 0.004  IFN-γ  r = 0.383  *P-*value = 0.010 |

r = Pearson correlation coefficient, calculated using SPSS Statistics 24®. A r-value close to 1 indicates a strong, positive correlation, whereas a r-value of -1 indicates a strong, negative correlation. *P*-values lower than 0.05 are considered to be significant.
