# Supplementary material for: In-feed bambermycin medication induces anti-inflammatory effects and prevents parietal cell loss without influencing Helicobacter suis colonization in the stomach of mice
Source: Vet Res. 2018 Apr 10;49:35. doi: 10.1186/s13567-018-0530-1 (PMC5894178; doi:10.1186/s13567-018-0530-1)
Supplement: Supplementary file 5 — Additional file 5. Overview of the relative fold changes of altered markers for inflammation in the bambermycin-supplemented and non-supplemented groups. The data are presented as fold changes in gene expression normalized to 3 reference genes and relative to control groups 1 and 4 (i.e. group 2-4 relative to group 1 and group 5-6 relative to group 4) which are considered as 1. The fold changes are shown as means with the standard error of the mean. Statistical differences were calculated using the non-parametric Kruskal–Wallis H test SPSS statistics 24®. A P-value lower than 0.05 is considered to be significant. Group 2 = 32 ppm bambermycin supplemented, non-H. suis infected group; group 3 = 64 ppm bambermycin supplemented, non-H. suis infected group; group 4 = H. suis-positive control group without bambermycin supplementation; group 5 = 32 ppm bambermycin supplemented, H. suis infected group; group 6 = 64 ppm bambermycin supplemented, H. suis infected group. [file 13567_2018_530_MOESM5_ESM.docx]

**Additional file 5**: Overview of the relative fold changes of altered markers for inflammation in the bambermycin-supplemented and non-supplemented groups.

| **Group** | **Gene** | **Relative fold change** | ***P*-value** |
| --- | --- | --- | --- |
| **2** |  |  |  |
|  | IL-1β | 0.71 ± 0.10 | 0.028 |
| **3** |  |  |  |
|  | IL-1β | 0.54 ± 0.10 | 0.015 |
| **4** |  |  |  |
|  | IL-4 | 2.37 ± 0.26 | 0.091 |
|  | IL-6 | 1.78 ± 0.38 | 0.115 |
|  | IL-8M | 2.65 ± 0.52 | 0.298 |
|  | IL-8Li | 5.18 ± 1.53 | 0.002 |
|  | IL-10 | 3.32 ± 1.07 | 0.012 |
|  | IL-17 | 0.13 ± 0.05 | <0.001 |
|  | IL-23 | 0.17 ± 0.03 | <0.001 |
|  | TNF-α | 2.69 ± 0.51 | 0.065 |
| **5** |  |  |  |
|  | IL-8M | 0.67 ± 0.22 | 0.100 |
|  | IFN-γ | 0.24 ± 0.08 | 0.015 |
| **6** |  |  |  |
|  | IL-8M | 0.23 ± 0.06 | 0.001 |
|  | IL-10 | 0.56 ± 0.06 | 0.077 |
|  | IFN-γ | 0.46 ± 0.20 | 0.253 |

The data are presented as fold changes in gene expression normalized to 3 reference genes and relative to control groups 1 and 4 (i.e. group 2-4 relative to group 1 and group 5-6 relative to group 4) which are considered as 1. The fold changes are shown as means with the standard error of the mean. Statistical differences were calculated using the non-parametric Kruskal-Wallis H test SPSS statistics 24®. A *P*-value lower than 0.05 is considered to be significant. Group 2 = 32 ppm bambermycin supplemented, non-*H. suis* infected group; group 3 = 64 ppm bambermycin supplemented, non-*H. suis* infected group; group 4 = *H. suis*-positive control group without bambermycin supplementation; group 5 = 32 ppm bambermycin supplemented, *H. suis* infected group; group 6 = 64 ppm bambermycin supplemented, *H. suis* infected group.
