# Supplementary material for: In-feed bambermycin medication induces anti-inflammatory effects and prevents parietal cell loss without influencing Helicobacter suis colonization in the stomach of mice
Source: Vet Res. 2018 Apr 10;49:35. doi: 10.1186/s13567-018-0530-1 (PMC5894178; doi:10.1186/s13567-018-0530-1)
Supplement: Supplementary file 6 — Additional file 6. Overview of the relative fold changes of altered markers for gastric acid secretion in the bambermycin-supplemented and non-supplemented groups. The data are presented as fold changes in gene expression normalized to 3 reference genes and relative to control groups 1 and 4 (i.e. group 2-4 relative to group 1 and group 5-6 relative to group 4) which are considered as 1. The fold changes are shown as means with the standard error of the mean. Statistical differences were calculated using the non-parametric Kruskal–Wallis H test SPSS statistics 24®. A P-value lower than 0.05 is considered to be significant. Group 2 = 32 ppm bambermycin supplemented, non-H. suis infected group; group 3 = 64 ppm bambermycin supplemented, non-H. suis infected group; group 4 = H. suis-positive control group without bambermycin supplementation; group 5 = 32 ppm bambermycin supplemented, H. suis infected group; group 6 = 64 ppm bambermycin supplemented, H. suis infected group. [file 13567_2018_530_MOESM6_ESM.docx]

**Additional file 6**: Overview of the relative fold changes of altered markers for gastric acid secretion in the bambermycin-supplemented and non-supplemented groups.

| **Group** | **Gene** | **Relative fold change** | ***P*-value** |
| --- | --- | --- | --- |
| 2 | KCNQ1 | 3.92 ± 0.87 | 0.023 |
|  | CCK-B receptor | 2.80 ± 0.80 | 0.214 |
|  | Gastrin | 0.68 ± 0.24 | 0.156 |
| 3 | H+/K ATPase | 2.22 ± 0.42 | 0.125 |
|  | Sonic Hedgehog | 2.01 ± 0.45 | 0.163 |
|  | KCNQ1 | 3.97 ± 0.81 | 0.014 |
|  | CCK-B receptor | 3.12 ± 0.49 | 0.035 |
|  | M3 receptor | 1.50 ± 0.17 | 0.181 |
| 4 | KCNQ1 | 3.24 ± 0.84 | 0.119 |
|  | CCK-B receptor | 3.46 ± 0.65 | 0.032 |
| 5 | CCK-B receptor | 0.67 ± 0.22 | 0.117 |
|  | Somatostatin | 0.63 ± 0.35 | 0.011 |

The data are presented as fold changes in gene expression normalized to 3 reference genes and relative to control groups 1 and 4 (i.e. group 2-4 relative to group 1 and group 5-6 relative to group 4) which are considered as 1. The fold changes are shown as means with the standard error of the mean. Statistical differences were calculated using the non-parametric Kruskal-Wallis H test SPSS statistics 24®. A *P*-value lower than 0.05 is considered to be significant. Group 2 = 32 ppm bambermycin supplemented, non-*H. suis* infected group; group 3 = 64 ppm bambermycin supplemented, non-*H. suis* infected group; group 4 = *H. suis*-positive control group without bambermycin supplementation; group 5 = 32 ppm bambermycin supplemented, *H. suis* infected group; group 6 = 64 ppm bambermycin supplemented, *H. suis* infected group.
