# Supplementary material for: In-feed bambermycin medication induces anti-inflammatory effects and prevents parietal cell loss without influencing Helicobacter suis colonization in the stomach of mice
Source: Vet Res. 2018 Apr 10;49:35. doi: 10.1186/s13567-018-0530-1 (PMC5894178; doi:10.1186/s13567-018-0530-1)
Supplement: Supplementary file 7 — Additional file 7. Overview of the number of pyrosequencing reads for each mice. Group 1 = H. suis-negative control group without bambermycin supplementation; group 2 = 32 ppm bambermycin supplemented, non-H. suis infected group; group 3 = 64 ppm bambermycin supplemented, non-H. suis infected group; group 4 = H. suis-positive control group without bambermycin supplementation; group 5 = 32 ppm bambermycin supplemented, H. suis infected group; group 6 = 64 ppm bambermycin supplemented, H. suis infected group. [file 13567_2018_530_MOESM7_ESM.docx]

**Additional file 7**: Overview of the number of pyrosequencing reads for each mice.

| **Mice** | **Group** | **Number of reads** |
| --- | --- | --- |
| M1_1 | 1 | 3776 |
| M1_2 | 1 | 2835 |
| M1_3 | 1 | 3771 |
| M1_7 | 1 | 3527 |
| M1_8 | 1 | 3806 |
| M2_1 | 2 | 3603 |
| M2_3 | 2 | 3020 |
| M2_5 | 2 | 3916 |
| M3_1 | 3 | 2586 |
| M3_5 | 3 | 3269 |
| M3_7 | 3 | 2861 |
| M3_8 | 3 | 2450 |
| M4_3 | 4 | 913 |
| M4_4 | 4 | 3004 |
| M4_5 | 4 | 1433 |
| M4_8 | 4 | 2430 |
| M5_2 | 5 | 3918 |
| M5_4 | 5 | 2839 |
| M5_7 | 5 | 3690 |
| M5_8 | 5 | 355 |
| M6_1 | 6 | 839 |
| M6_4 | 6 | 3734 |
| M6_6 | 6 | 411 |
| M6_7 | 6 | 3219 |
| M6_8 | 6 | 3895 |

Group 1 = *H. suis*-negative control group without bambermycin supplementation; group 2 = 32 ppm bambermycin supplemented, non-*H. suis* infected group; group 3 = 64 ppm bambermycin supplemented, non-*H. suis* infected group; group 4 = *H. suis*-positive control group without bambermycin supplementation; group 5 = 32 ppm bambermycin supplemented, *H. suis* infected group; group 6 = 64 ppm bambermycin supplemented, *H. suis* infected group.
