# Supplementary material for: In-feed bambermycin medication induces anti-inflammatory effects and prevents parietal cell loss without influencing Helicobacter suis colonization in the stomach of mice
Source: Vet Res. 2018 Apr 10;49:35. doi: 10.1186/s13567-018-0530-1 (PMC5894178; doi:10.1186/s13567-018-0530-1)
Supplement: Supplementary file 10 — Additional file 10. An overview of the main differences in relative abundance of taxa at phylum, family, genus and species level in the bambermycin-supplemented and non-supplemented groups. The data are presented as the mean relative abundance of the taxa with the standard error of the mean. Statistical differences were calculated using the non-parametric Kruskal–Wallis tests with Tukey post hoc tests and Benjamini–Hochberg False Discovery Rate were performed using STAMP®. A P-value lower than 0.05 is considered to be significant. [file 13567_2018_530_MOESM10_ESM.docx]

**Additional file 10**: An overview of the main differences in relative abundance of taxa at phylum, family, genus and species level in the bambermycin-supplemented and non-supplemented groups.

| **Level** | **Taxa** | **Bambermycin +** | **Bambermycin -** | ***P*-value** |
| --- | --- | --- | --- | --- |
| **Phylum** | Bacteria_unclassified | 0*.*00 ± 0.00 | 0*.*12 ± 0.06 | 0*.*025 |
| **Family** | Firmicutes_unclassified | 0*.*05 ± 0.02 | 0*.*64 ± 0.26 | 0*.*048 |
|  | Clostridiaceae_1 | 3*.*07 ± 1.37 | 24*.*08 ± 10.30 | 0*.*300 |
|  | Bacteria_unclassified | 0*.*00 ± 0.00 | 0*.*12 ± 0.06 | 0*.*048 |
| **Genus** | *Clostridiaceae_*1_unclassified | 0*.*04 ± 0.03 | 0*.*23 ± 0.11 | 0*.*445 |
|  | *Firmicutes*_unclassified | 0*.*05 ± 0.02 | 0*.*64 ± 0.26 | 0*.*024 |
|  | *Turicibacter* | 0*.*00 ± 0.00 | 3*.*12 ± 1.39 | 0*.*018 |
|  | *Clostridium*_sensu_stricto_1 | 3*.*02 ± 1.34 | 23*.*85 ± 10.25 | 0*.*243 |
|  | Bacteria_unclassified | 0*.*00 ± 0.00 | 0*.*12 ± 0.06 | 0*.*018 |
|  | *Coprococcus* | 0*.*00 ± 0.00 | 0*.*11 ± 0.05 | 0*.*014 |
| **Species** | *Coprococcus*_EF099198 | 0*.*00 ± 0.00 | 0*.*49 ± 0.44 | 0*.*021 |
|  | *Coprococcus*_16S_OTU119 | 0*.*00 ± 0.00 | 0*.*07 ± 0.03 | 0*.*031 |
|  | *Clostridiales*_Family_XIII_AB702776 | 0*.*00 ± 0.00 | 0*.*17 ± 0.15 | 0*.*023 |
|  | *Clostridiales_*Family_XIII_16S_OTU162 | 0*.*00 ± 0.00 | 0*.*07 ± 0.05 | 0*.*035 |
|  | *Clostridiales*_Family_XIII_EF604613 | 0*.*00 ± 0.00 | 0*.*48 ± 0.41 | 0*.*131 |
|  | *Clostridiaceae*_1_16S_OTU75 | 0*.*00 ± 0.00 | 0*.*08 ± 0.05 | 0*.*033 |
|  | *Clostridiaceae*_1_16S_OTU107 | 0*.*01 ± 0.01 | 0*.*05 ± 0.03 | 0*.*469 |
|  | *Firmicutes*_16S_OTU195 | 0*.*00 ± 0.00 | 0*.*01 ± 0.01 | 0*.*035 |
|  | *Firmicutes*_16S_OTU37 | 0*.*00 ± 0.00 | 0*.*14 ± 0.07 | 0*.*039 |
|  | *Firmicutes*_16S_OTU43 | 0*.*00 ± 0.00 | 0*.*18 ± 0.09 | 0*.*090 |
|  | *Firmicutes*_16S_OTU594 | 0*.*00 ± 0.00 | 0*.*02 ± 0.01 | 0*.*126 |
|  | *Turicibacter*_EF406660 | 0*.*00 ± 0.00 | 3*.*96 ± 2.12 | 0*.*058 |
|  | *Turicibacter*_DQ015666 | 0*.*00 ± 0.00 | 0*.*02 ± 0.01 | 0*.*138 |
|  | *Turicibacter*_EF406615 | 0*.*00 ± 0.00 | 0*.*08 ± 0.06 | 0*.*104 |
|  |  | ***H. suis* +** | ***H. suis* -** | ***P*-value** |
| **Species** | *Christensenella*_EF603775 | 0*.*00 ± 0.00 | 0*.*041 ± 0.019 | 0*.*223 |

The data are presented as the mean relative abundance of the taxa with the standard error of the mean. Statistical differences were calculated using the non-parametric Kruskal-Wallis tests with Tukey post-hoc tests and Benjamini-Hochberg False Discovery Rate were performed using STAMP®. A *P*-value lower than 0.05 is considered to be significant.
