# Supplementary material for: In-feed bambermycin medication induces anti-inflammatory effects and prevents parietal cell loss without influencing Helicobacter suis colonization in the stomach of mice
Source: Vet Res. 2018 Apr 10;49:35. doi: 10.1186/s13567-018-0530-1 (PMC5894178; doi:10.1186/s13567-018-0530-1)
Supplement: Supplementary file 11 — Additional file 11. Overview of important correlations between the gastric bacterial community and the number of inflammatory cells, parietal cells and expression of markers for inflammation and gastric acid secretion. r = Pearson correlation coefficient, calculated using SPSS Statistics 24®. A r-value close to 1 indicates a strong, positive correlation, whereas a r-value of -1 indicates a strong, negative correlation. P-values lower than 0.05 are considered to be significant. [file 13567_2018_530_MOESM11_ESM.docx]

**Additional file 11**: Overview of important correlations between the gastric bacterial community and the number of inflammatory cells, parietal cells and expression of markers for inflammation and gastric acid secretion

|  | **Taxa** | **Expression of markers for inflammation, gastric acid secretion and number of infiltrating cells** |
| --- | --- | --- |
| **Family** | Firmicutes_unclassified | Gastrin  r = 0.672  *P-*value < 0.001 |
|  |  | Infiltration with inflammatory cells  r = 0.444  *P-*value = 0.026 |
|  |  | Parietal cells  r = -0.414  *P-*value = 0.040 |
|  |  | T-cells  r = 0.529  *P-*value = 0.007 |
|  |  | B-cells  r = 0.561  *P-*value = 0.003 |
|  | Clostridiaceae_1 | Gastrin  r = 0.538  *P-*value = 0.007 |
|  |  | IFNg  r = 0.556  *P-*value = 0.009 |
|  |  | Parietal cells  r = -0.516  *P-*value = 0.008 |
|  |  | B-cells  r = 0.447  *P-*value = 0.025 |
|  | Bacteria_unclassified | Gastrin  r = 0.398  *P-*value = 0.054 |
|  |  | Infiltration with inflammatory cells  r = 0.356  *P-*value = 0.080 |
|  |  | T-cells  r = 0.472  *P-*value = 0.017 |
|  |  | B-cells  r = 0.362  *P-*value = 0.075 |
| **Genus** | *Firmicutes*_unclassified | Gastrin  r = 0.672  *P-*value = 0 |
|  |  | IFNg  r = 0.432  *P-*value = 0.050 |
|  |  | Infiltration with inflammatory cells  r = 0.444  *P-*value = 0.026 |
|  |  | Parietal cells  r = -0.414  *P-*value = 0.040 |
|  |  | T-cells  r = 0.529  *P-*value = 0.007 |
|  |  | B-cells  r = 0.561  *P-*value = 0.003 |
|  | *Clostridiaceae*_1_unclassified | Gastrin  r = 0.526  *P-*value = 0.008 |
|  |  | Somatostatin  r = 0.351  *P-*value = 0.085 |
|  |  | IFNg  r = 0.565  *P-*value = 0.008 |
|  |  | Parietal cells  r = -0.510  *P-*value = 0.009 |
|  |  | B-cells  r = 0.513  *P-*value = 0.009 |
|  | *Clostridium*_sensu_stricto_1 | Gastrin  r = 0.538  *P-*value = 0.007 |
|  |  | IFNg  r = 0.556  *P-*value = 0.009 |
|  |  | Parietal cells  r = -0.516  *P-*value = 0.008 |
|  |  | B-cells  r = 0.447  *P-*value = 0.025 |
|  | Bacteria_unclassified | Gastrin  r = 0.398  *P-*value = 0.054 |
|  |  | Infiltration with inflammatory cells  r = 0.356  *P-*value = 0.080 |
|  |  | T-cells  r = 0.472  *P-*value = 0.017 |
|  |  | B-cells  r = 0.362  *P-*value = 0.075 |
|  | *Turicibacter* | Gastrin  r = 0.548  *P-*value = 0.006 |
|  |  | IFNg  r = 0.403  *P-*value = 0.070 |
|  |  | Infiltration with inflammatory cells  r = 0.599  *P-*value = 0.002 |
|  |  | T-cells  r = 0.741  *P-*value < 0.001 |
|  |  | B-cells  r = 0.389  *P-*value = 0.054 |
|  |  | Marcophages  r = 0.505  *P-*value = 0.01 |
|  | *Coprococcus* | KCNQ1  r = -0.502  *P-*value = 0.015 |
|  |  | Gastrin  r = 0.391  *P-*value = 0.059 |
|  |  | H2Rc  r = -0.450  *P-*value = 0.024 |
|  |  | Infiltration with inflammatory cells  r = 0.621  *P-*value = 0.001 |
|  |  | T-cells  r = 0.600  *P-*value = 0.002 |
|  |  | B-cells  r = 0.347  *P-*value = 0.09 |
| **Species** | *Coprococcus*_EF099198 | KCNQ1  r = -0.441  *P-*value = 0.035 |
|  |  | H2Rc  r = -0.372  *P-*value = 0.067 |
|  |  | Infiltration with inflammatory cells  r = 0.555  *P-*value = 0.004 |
|  |  | T-cells  r = 0.485  *P-*value = 0.014 |
|  |  | B-cells  r = 0.374  *P-*value = 0.066 |
|  | *Coprococcus*_16S_OTU119 | KCNQ1  r = -0.506  *P-*value = 0.014 |
|  |  | Gastrin  r = 0.401  *P-*value = 0.052 |
|  |  | H2Rc  r = -0.458  *P-*value = 0.021 |
|  |  | Infiltration with inflammatory cells  r = 0.621  *P-*value = 0.001 |
|  |  | T-cells  r = 0.620  *P-*value = 0.001 |
|  | *Clostridiales*_Family_XIII_AB702776 | Infiltration with inflammatory cells  r = 0.486  *P-*value = 0.014 |
|  |  | T-cells  r = 0.469  *P-*value = 0.018 |
|  |  | B-cells  r = 0.381  *P-*value = 0.060 |
|  |  | Macrophages  r = 0.412  *P-*value = 0.041 |
|  | *Clostridiales*_Family_XIII_16S_OTU162 | H+/K+ ATPase  r = -0.417  *P-*value = 0.038 |
|  |  | KCNQ1  r = -0.458  *P-*value = 0.028 |
|  |  | H2Rc  r = -0.448  *P-*value = 0.025 |
|  |  | Infiltration with inflammatory cells  r = 0.486  *P-*value = 0.014 |
|  |  | T-cells  r = 0.416  *P-*value = 0.039 |
|  |  | B-cells  r = 0.381  *P-*value = 0.060 |
|  | *Clostridiales*_Family_XIII_EF604613 | H+/K+ ATPase  r = -0.367  *P-*value = 0.071 |
|  |  | Infiltration with inflammatory cells  r = 0.412  *P-*value = 0.041 |
|  |  | T-cells  r = 0.395  *P-*value = 0.051 |
|  |  | B-cells  r = 0.386  *P-*value = 0.057 |
|  | *Clostridiaceae*_1_16S_OTU75 | Gastrin  r = 0.355  *P-*value = 0.088 |
|  |  | Infiltration with inflammatory cells  r = 0.35  *P-*value = 0.087 |
|  |  | T-cells  r = 0.506  *P-*value = 0.010 |
|  |  | B-cells  r = 0.495  *P-*value = 0.012 |
|  |  | Macrophaes  r = 0.495  *P-*value = 0.012 |
|  | *Clostridiaceae*_1_16S_OTU107 | IFNg  r = 0.468  *P-*value = 0.032 |
|  |  | Parietal cells  r = -0.581  *P-*value = 0.002 |
|  |  | B-cells  r = 0.466  *P-*value = 0.019 |
|  |  | Macrophages  r = 0.507  *P-*value = 0.010 |
|  | *Firmicutes*_16S_OTU195 | Gastrin  r = 0.437  *P-*value = 0.033 |
|  |  | IFNg  r = 0.373  *P-*value = 0.096 |
|  |  | Infiltration with inflammatory cells  r = 0.555  *P-*value = 0.001 |
|  |  | T-cells  r = 0.455  *P-*value = 0.022 |
|  |  | Macrophages  r = 0.355  *P-*value = 0.081 |
|  | *Firmicutes*_16S_OTU37 | Gastrin  r = 0.512  *P-*value = 0.011 |
|  |  | Infiltration with inflammatory cells  r = 0.685  *P-*value < 0.001 |
|  |  | T-cells  r = 0.665  *P-*value < 0.001 |
|  |  | B-cells  r = 0.347  *P-*value = 0.090 |
|  |  | Macrophages  r = 0.439  *P-*value = 0.028 |
|  | *Firmicutes*_16S_OTU43 | Gastrin  r = 0.428  *P-*value = 0.037 |
|  |  | H2Rc  r = -0.349  *P-*value = 0.088 |
|  |  | IFNg  r = 0.470  *P-*value = 0.032 |
|  |  | Parietal cells  r = -0.371  *P-*value = 0.068 |
|  |  | B-cells  r = 0.557  *P-*value = 0.004 |
|  | *Firmicutes*_16S_OTU594 | H+/K+ ATPase  r = -0.348  *P-*value = 0.088 |
|  |  | H2Rc  r = -0.362  *P-*value = 0.075 |
|  |  | Infiltration with inflammatory cells  r = 0.412  *P-*value = 0.041 |
|  |  | T-cells  r = 0.389  *P-*value = 0.055 |
|  |  | B-cells  r = 0.369  *P-*value = 0.069 |
|  |  | Macrophages  r = 0.339  *P-*value = 0.098 |
|  | *Turicibacter*_EF406660 | Gastrin  r = 0.548  *P-*value = 0.006 |
|  |  | IFNg  r = 0.403  *P-*value = 0.070 |
|  |  | Infiltration with inflammatory cells  r = 0.599  *P-*value = 0.002 |
|  |  | T-cells  r = 0.741  *P-*value < 0.001 |
|  |  | B-cells  r = 0.389  *P-*value = 0.054 |
|  |  | Macrophages  r = 0.505  *P-*value = 0.010 |
|  | *Turicibacter*_DQ015666 | H+/K+ ATPase  r = -0.347  *P-*value = 0.090 |
|  |  | H2Rc  r = -0.365  *P-*value = 0.073 |
|  |  | Infiltration with inflammatory cells  r = 0.412  *P-*value = 0.041 |
|  |  | T-cells  r = 0.384  *P-*value = 0.058 |
|  |  | B-cells  r = 0.364  *P-*value = 0.074 |
|  | *Turicibacter*_EF406615 | KCNQ1  r = -0.395  *P-*value = 0.062 |
|  |  | Gastrin  r = 0.387  *P-*value = 0.062 |
|  |  | H2Rc  r = -0.469  *P-*value = 0.018 |
|  |  | Infiltration with inflammatory cells  r = 0.486  *P-*value = 0.014 |
|  |  | T-cells  r = 0.530  *P-*value = 0.006 |
|  | *Christensenella*_EF603775 | IL-6  r = -0.490  *P-*value = 0.015 |
|  |  | IL-17  r = 0.362  *P-*value = 0.082 |
|  |  | IL-23  r = 0.452  *P-*value = 0.023 |
|  |  | TNF-α  r = -0.423  *P-*value = 0.040 |

r = Pearson correlation coefficient, calculated using SPSS Statistics 24®. A r-value close to 1 indicates a strong, positive correlation, whereas a r-value of -1 indicates a strong, negative correlation. *P-*values lower than 0.05 are considered to be significant.
